# Uropathogenic *E*. *coli* Exploit CEA to Promote Colonization of the Urogenital Tract Mucosa

**DOI:** 10.1371/journal.ppat.1005608

**Published:** 2016-05-12

**Authors:** Petra Muenzner, Arnaud Kengmo Tchoupa, Benedikt Klauser, Thomas Brunner, Johannes Putze, Ulrich Dobrindt, Christof R. Hauck

**Affiliations:** 1 Lehrstuhl Zellbiologie, Fachbereich Biologie, Universität Konstanz, Konstanz, Germany; 2 Lehrstuhl Biochemische Pharmakologie, Fachbereich Biologie, Universität Konstanz, Konstanz, Germany; 3 Institut für Hygiene, Universität Münster, Münster, Germany; 4 Konstanz Research School Chemical Biology, Universität Konstanz, Konstanz, Germany; University of Illinois, UNITED STATES

## Abstract

Attachment to the host mucosa is a key step in bacterial pathogenesis. On the apical surface of epithelial cells, members of the human carcinoembryonic antigen (CEA) family are abundant glycoproteins involved in cell-cell adhesion and modulation of cell signaling. Interestingly, several gram-negative bacterial pathogens target these receptors by specialized adhesins. The prototype of a CEACAM-binding pathogen, *Neisseria gonorrhoeae*, utilizes colony opacity associated (Opa) proteins to engage CEA, as well as the CEA-related cell adhesion molecules CEACAM1 and CEACAM6 on human epithelial cells. By heterologous expression of neisserial Opa proteins in non-pathogenic *E*. *coli* we find that the Opa protein-CEA interaction is sufficient to alter gene expression, to increase integrin activity and to promote matrix adhesion of infected cervical carcinoma cells and immortalized vaginal epithelial cells *in vitro*. These CEA-triggered events translate in suppression of exfoliation and improved colonization of the urogenital tract by Opa protein-expressing *E*. *coli* in CEA-transgenic compared to wildtype mice. Interestingly, uropathogenic *E*. *coli* expressing an unrelated CEACAM-binding protein of the Afa/Dr adhesin family recapitulate the *in vitro* and *in vivo* phenotype. In contrast, an isogenic strain lacking the CEACAM-binding adhesin shows reduced colonization and does not suppress epithelial exfoliation. These results demonstrate that engagement of human CEACAMs by distinct bacterial adhesins is sufficient to blunt exfoliation and to promote host infection. Our findings provide novel insight into mucosal colonization by a common UPEC pathotype and help to explain why human CEACAMs are a preferred epithelial target structure for diverse gram-negative bacteria to establish a foothold on the human mucosa.

## Author Summary

Mucous surfaces are a hallmark of the nasal cavity and the throat as well as the intestinal and urogenital tracts. These surfaces serve as primary entry portals for a large number of pathogenic bacteria. To get a foothold on the mucosa, bacteria not only need to tightly attach to this tissue, but also need to overcome an intrinsic defence mechanism called exfoliation. During the exfoliation process, the outermost cell layer, together with attached bacteria, is released from the tissue surface reducing the microbial burden. A comprehensive understanding of the molecular strategies, which bacteria utilize to undermine this host defence, is currently lacking. Our results suggest that different bacterial pathogens have found a surprisingly similar answer to this problem by targeting a common set of proteins on the tissue surface. Accordingly, these bacteria express unrelated proteins that engage the same host receptors called CEA-related cell adhesion molecules (CEACAMs). Binding of microbes to CEACAMs triggers, via intracellular signaling pathways, an increased stickiness of the infected cells. Thereby, the pathogens suppress the release of superficial host cells from the tissue and effectively block exfoliation. Detailed mechanistic insight into this process and the ability to manipulate exfoliation might help to prevent or treat bacterial infections.

## Introduction

During evolution bacteria have developed fascinating strategies to colonize multicellular organisms. A first critical step, which in many cases determines the outcome of the microbe-host encounter, is the ability of the microorganisms to establish themselves on mucosal surfaces [1,2]. Attachment to the mucosa is facilitated by specific bacterial adhesins, which firmly connect the microbe to the tissue [3,4]. Indeed, adhesin-mediated bacteria-host interactions prevent mechanical removal of the microbes via mucociliary cleansing or urinary flow, and can be seen as a prerequisite for efficient colonization. However, mucosal epithelia have several additional tissue-intrinsic defense mechanisms that protect the surface from adherent pathogens [5]. For example, in both stratified as well as single-layered epithelia the superficial cells are constantly replaced from a stem cell population. This tissue turnover also leads to shedding of cell-associated microbes from the epithelium reducing the bacterial burden. Epithelial tissue turnover can be very fast, as in the intestinal epithelium, where the superficial cells on the exposed villus folds are continuously replaced every day and where this process helps to maintain intestinal homeostasis. Indeed, slowing down tissue turnover in the intestinal tract can facilitate pathogen colonization [6,7]. Similar to the single-layered epithelium of the gut, stratified epithelia of the urogenital tract are also subject to continuous tissue renewal, albeit at a lower rate. However, exposure to high numbers of bacteria can trigger an accelerated turnover, whereby large amounts of superficial epithelial cells are released, a mechanism also known as exfoliation [8–12]. Exfoliation is an innate protective mechanism that, via rapid detachment and shedding of the infected superficial cells, limits colonization of the tissue by the microflora and ultimately prohibits further penetration of the bacteria [13]. By this process, even cell-associated bacteria can be removed from the tissue surface together with the infected cells.

Recently, we could show that specialized bacteria, which colonize the human urogenital tract, are able to suppress the exfoliation response [14]. These bacteria utilize outer membrane adhesins, the so-called Opa_CEA_ proteins, to bind to members of the CEACAM family, a group of immunoglobulin-related gylcoproteins expressed on the apical membrane of mucosal epithelial cells (for review see [15]). CEACAM engagement by bacteria triggers activation of integrins, enhances matrix adhesion and reduces cell detachment of infected cells, ultimately facilitating bacterial colonization [14,16]. Besides *Neisseria gonorrhoeae*, which was studied in these previous investigations, also the closely related *Neisseria meningitidis* expresses CEACAM-binding Opa proteins and exploits mucosal CEACAMs such as CEACAM1 or CEA (the product of the *CEACAM5* gene) for contacting host cells and for colonization of the nasopharynx [17–19]. In both instances, the pathogens selectively bind to human, but not other mammalian CEACAM family members [20], suggesting that this exquisite recognition mechanism is a result of the co-evolution of these microbes with their sole natural host. Indeed, by interfering with epithelial exfoliation Opa_CEA_-expressing *Neisseria* should have a clear advantage during colonization of the human mucosa. It is currently unclear if the expression of Opa_CEA_ proteins is sufficient to counteract exfoliation or if additional neisserial virulence factors are involved in this process. This aspect is of particular interest, as several unrelated bacterial pathogens, such as *Haemophilus influenzae*, *Moraxella catarrhalis*, and pathogenic strains of *Escherichia coli*, have been shown to possess distinct CEACAM-binding adhesins (for an overview see [21]) and might trigger similar processes.

To address this question, we have investigated if engagement of human CEACAMs by a bacterial CEACAM-binding adhesin is sufficient to counteract exfoliation and to promote mucosal colonization. Indeed, we find that expression of a neisserial Opa_CEA_ protein in non-pathogenic *E*. *coli* allows these bacteria to engage CEA on epithelial cells, to trigger increased integrin activity and cell-matrix adhesion, and to promote mucosal colonization. Importantly, uropathogenic *E*. *coli* (UPEC), harboring a CEACAM-binding adhesin of the Afa/Dr family, also exploit this mechanism to block epithelial exfoliation and to boost their ability to colonize the urogenital tract *in vivo*. Together, our results establish human CEACAMs as a preferred epithelial target structure, which allows diverse gram-negative bacterial pathogens to suppress exfoliation and to efficiently colonize the human mucosa.

## Results

### A CEACAM-binding adhesin is sufficient to counteract the detachment of human cervical epithelial cells upon bacterial infection

**Fig 1:**
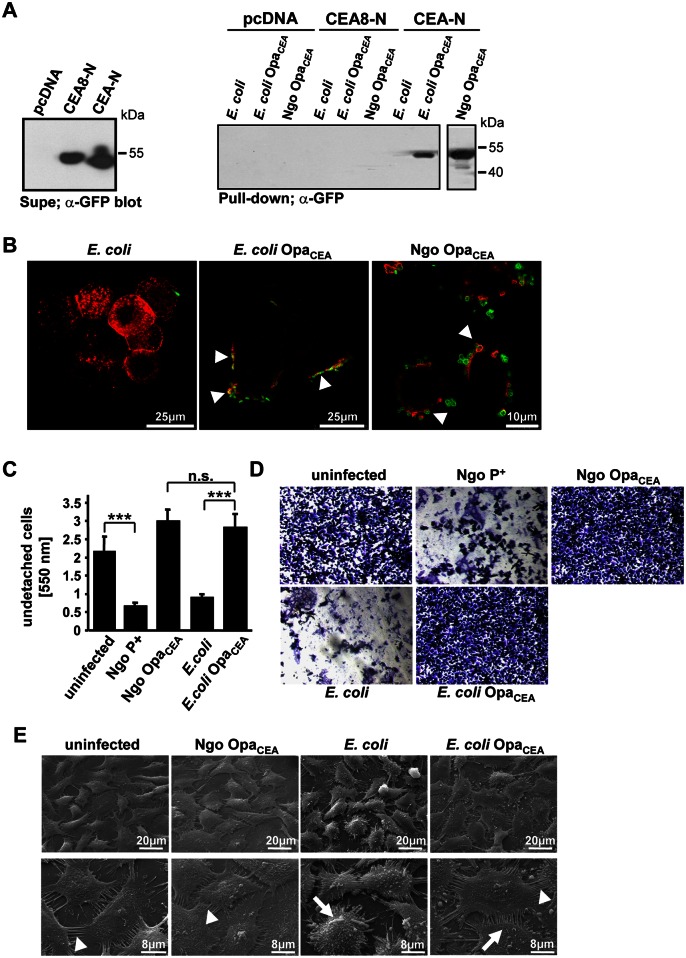
CEACAM engagement by *E*. *coli* strains expressing Opa_CEA_ adhesins blocks the detachment of human cervical epithelial cells. *(A)* Cell culture supernatants containing GFP-fused Ig_V_-like domains of CEA (CEA-N) or CEACAM8 (CEA8-N) or control supernatants (pcDNA) were prepared from transfected 293 cells and analysed by Western blotting with a monoclonal anti-GFP antibody to verify similar amounts of the soluble fusion proteins (Supe; left panel). *E*. *coli*, *E*. *coli* expressing a CEACAM-binding Opa protein from *N*. *gonorrhoeae* (*E*. *coli* Opa_CEA_), or non-piliated, Opa_CEA_-expressing *N*. *gonorrhoeae* MS11 (Ngo Opa_CEA_) were incubated with the indicated CEA-N- or CEA8-N-containing or control cell culture supernatants for 2 h at room temperature. Bacteria were washed with PBS and bacteria-bound GFP-fusion proteins were detected by Western blotting with anti-GFP antibody (Pull-down; right panel). Shown is a representative experiment out of three independent biological replicates. *(B)* ME-180 cells were seeded on glasscover slips, infected with the indicated bacteria for 2 h, fixed and co-stained with a monoclonal antibody against CEACAMs (clone D14HD11; red) together with polyclonal rabbit antibodies against *E*. *coli* or against *N*. *gonorrhoeae* MS11 (green). Presented are merged images showing the recruitment of CEACAMs to cell-associated CEACAM-binding bacteria (arrowheads). Pictures are representative for four independent biological replicates. *(C)* ME-180 cells were seeded on 25 μg/ml collagen, confluent layers were left uninfected or infected for 14h with piliated, non-opaque *N*. *gonorrhoeae* (Ngo P+), Ngo Opa_CEA_, *E*. *coli*, or *E*. *coli* Opa_CEA_. Following infection, cells were washed and remaining cells were stained with crystal violet. Staining intensity was determined after dye elution in a spectrophotometer at 550 nm. Bars represent mean ± SD of eight replicates. Two-tailed student’s t-test; *** p < 0.001, n.s.—not significant. Shown is one representative experiment out of four independent biological replicates. *(D)* ME-180 cells were infected and stained as in (C). Representative areas with remaining cells were photographed. *(E)* Confluent layers of ME-180 cultures were infected with the indicated bacteria or left uninfected. After 14 h, cells were fixed *in situ* and analysed by scanning electron microscopy. Uninfected ME-180 cells were tightly adherent to the cell culture surface and displayed numerous cell-cell contacts (arrowheads). In contrast, cells infected with *E*. *coli* rounded up and reduced cell-cell contacts, whereas cells infected with Opa_CEA_-expressing *E*. *coli* or *N*. *gonorrhoeae* retained cell-cell contacts (arrowheads) despite the presence of bacteria. Pictures are representative for four independent biological replicates.

Previous work demonstrated that a CEACAM-binding adhesin is necessary to allow *Neisseria gonorrhoeae* to increase extracellular matrix binding of infected cells [14,16]. To test if CEACAM-binding alone is sufficient to trigger this process, we separated the CEACAM-binding Opa_CEA_ adhesin from other gonococcal virulence determinants by expressing this neisserial outer membrane protein in *E*. *coli*. In the used expression plasmid pTrc, Opa_CEA_-protein expression is under control of the IPTG-inducible *lac* promoter [22]. Due to the leakiness of this promoter, Opa_CEA_ protein-expression was already observed under non-inducing conditions in Opa_CEA_-expressing *E*. *coli* (*E*. *coli* Opa_CEA_) compared to an *E*. *coli* strain harbouring the empty pTrc plasmid (S1A Fig). The Opa_CEA_ protein expressed in *E*. *coli* showed a similar size to the native Opa_CEA_ protein expressed in *N*. *gonorrhoeae*, but levels were only about 20% of that observed in gonococci (S1A Fig). Both *E*. *coli* strains showed a comparable growth pattern in liquid culture, indicating that expression of the Opa_CEA_ protein in *E*. *coli* at this level did not interfere with growth (S1B Fig). In contrast, strong overexpression, e.g. upon IPTG induction, can retard *E*. *coli* growth (S1C Fig). When expressed in *E*. *coli*, the neisserial Opa protein adhesin was functional with regard to CEACAM-binding, as *E*. *coli* Opa_CEA_ was able to associate with the GFP-tagged amino-terminal domain of CEA (CEA-N; Fig 1A). The control *E*. *coli* strain did not bind to soluble CEA-N, and no binding of either *E*. *coli* strain to the CEACAM8-amino-terminal domain (CEA8-N) was observed (Fig 1A). This binding pattern of the heterologously expressed Opa_CEA_ protein is in agreement with the CEACAM binding profile observed for *N*. *gonorrhoeae* expressing this Opa_CEA_ protein (Fig 1A). To investigate, whether *E*. *coli* Opa_CEA_ interacts with CEA in a cellular context, we infected the human cervical epithelial cell line ME-180 for 2 h with the *E*. *coli* control strain or with *E*. *coli* Opa_CEA_. ME-180 cells endogenously express CEACAM1, CEACAM6, and CEA, which were located in the plasma membrane of uninfected cells (S2A and S2B Fig). Upon infection with the *E*. *coli* control strain, the distribution of CEACAMs was unchanged and these bacteria did not associate with ME-180 cells (Fig 1B and S2B Fig). Importantly, *E*. *coli* Opa_CEA_ strongly adhered to ME-180 cells and triggered re-location and local concentration of CEACAMs at sites of bacteria-host cell contact (Fig 1B and S2B Fig; arrowheads). Similarly, Opa_CEA_ protein-expressing gonococci adhered in large numbers to ME-180 cells and induced CEACAM clustering (Fig 1B and S2B Fig). Together, these results demonstrated that the neisserial adhesin is functionally expressed in *E*. *coli*, where it promotes interaction with CEA-expressing cells.

CEA engagement by pathogenic *N*. *gonorrhoeae* has been shown to trigger increased matrix adhesion and to counteract the detachment of epithelial cells [14]. Therefore, we infected confluent monolayers of ME-180 cells, grown on collagen, for 14 h or left the ME-180 cells uninfected. Next, monolayers were washed to remove detached and loosely adherent cells and the remaining cells were stained with crystal violet. Elution and quantification of the dye in a spectrophotometer served as a measure of the remaining adherent cells. Interestingly, in samples infected with Opa_CEA_ protein-expressing *N*. *gonorrhoeae* as well as in samples infected with Opa_CEA_ protein-expressing *E*. *coli* no reduction in the amount of adherent cells compared to uninfected cells was observed (Fig 1C). Indeed, ME-180 cells infected with CEACAM-binding bacteria even showed a slightly higher recovery than uninfected cells suggesting that cell-matrix adhesion is reinforced upon infection with Opa_CEA_ protein expressing bacteria (Fig 1C). In contrast to CEACAM-binding bacteria, cells infected with the *E*. *coli* control strain or with piliated, non-CEACAM-binding gonococci (Ngo P+) showed pronounced detachment of cells (Fig 1C). Microscopic observation of the infected cell cultures corroborated the massive loss of epithelial cells from monolayers infected with control *E*. *coli* or Ngo P+, whereas cell numbers in samples infected for prolonged times with Opa_CEA_ protein-expressing bacteria were even higher than in uninfected samples (Fig 1D). Similar results were obtained, when primary human vaginal epithelial (hVECs) cells were infected for 14 h with either Opa_CEA_ protein-expressing bacteria or non-CEACAM-binding strains. Again, expression of a CEACAM-binding adhesin was able to block the infection-induced detachment of the primary epithelial cells (S3A and S3B Fig). Scanning electron microscopy revealed that ME-180 cells infected for 14 h with control *E*. *coli* reduced cell-cell-contacts and rounded up, whereas cells infected with Opa_CEA_ protein-expressing *E*. *coli* remained well-spread on collagen similar to uninfected cultures and similar to cells infected with Opa_CEA_-protein expressing gonococci (Fig 1E). These results demonstrate that CEACAM engagement via a CEACAM-binding adhesin, even when expressed in a heterologous background, is sufficient to counteract the detachment of infected epithelial cells *in vitro*.

### *E*. *coli* Opa_CEA_ engagement of CEA leads to upregulation of CD105 expression, increased cell adhesion, and integrin activation

Both human primary vaginal epithelial cells as well as ME180 cervical carcinoma cells endogenously express members of the CEACAM family, in including CEA (S2B and S3C Figs). To rigorously test the contribution of CEACAMs to this process, we employed 293 cells, a human cell line that lacks endogenous CEACAM expression. Upon transient transfection with a CEA-encoding plasmid, about 40% of the cell population showed surface expression of the receptor (S4A Fig). Control transfected or CEA-expressing cells were then infected for 5 h with the indicated bacterial strains and used in cell adhesion assays on collagen. We again observed increased matrix binding of CEA-expressing cells upon infection with Opa_CEA_ protein-expressing *E*. *coli* (S4B Fig). The adhesion of CEA-expressing cells infected with *E*. *coli* Opa_CEA_ was comparable to the increased cell adhesion seen upon infection with Opa_CEA_ protein-expressing gonococci. In contrast, incubation with the *E*. *coli* control strain did not alter matrix adhesion of the infected cells (S4B Fig). Importantly, 293 cells transfected with an empty control plasmid (pcDNA) did not display changes in adhesiveness, irrespective of the bacterial strain used for infection (S4B Fig). These results demonstrate that it is the adhesin-CEACAM interaction, which serves as the trigger for increased extracellular matrix adhesion of the infected cells.

Previously, CEACAM engagement by bacteria has been shown to induce CD105 expression in epithelial cells, which was a pre-requisite for enhanced matrix adhesion of infected cells [16]. Indeed, infection of CEA-expressing 293 cells with Opa_CEA_ protein-expressing *E*. *coli* or *N*. *gonorrhoeae* resulted in the presence of CD105 on the surface of the cells, whereas uninfected cells or cells infected with control *E*. *coli* did not have detectable CD105 on their surface (S4C Fig). In line with the idea that CEACAM-triggered expression of CD105 is critical for increased cell adhesion, expression of CD105 in 293 cells led to strongly elevated cell-matrix adhesion in the absence of bacterial infection or CEACAM stimulation (S4B Fig).

Though bacterial infection or CD105 expression resulted in alterations in cell-matrix adhesion, the amount of surface exposed integrin β1 was unaltered in all infected samples compared to the uninfected control (S5A Fig). However, infection with CEACAM-binding gonococci or Opa_CEA_ protein-expressing *E*. *coli* promoted a conformational change of integrin β1 as detected by the conformation-sensitive anti-integrin β1 antibody 9EG7 (S5B Fig). Increased labeling by antibody 9EG7 indicated that CEA-engagement by bacteria led to pronounced activation of integrins. In contrast, integrin activity remained low in uninfected cells or cells infected with non-CEACAM-binding bacteria (S5B Fig). The increased integrin activity clearly depended on the presence of CEA, as 293 cells transfected with the empty control vector did not alter the amount of surface integrin nor integrin activity upon infection with diverse bacteria (S5C and S5D Fig). As a further control, cells were stimulated with Mn^2+^, an exogenous activator of integrins. Upon Mn^2+^ addition, a similar, maximal integrin activity was observed in all samples demonstrating that the total activatable integrin levels on the cell surface were similar (S5B and S5D Fig). Together, these findings imply that *E*. *coli* Opa_CEA_, via engagement of CEACAMs, can trigger CD105 expression, which in turn enhances integrin activity *in vitro*.

### The CEA–*E*. *coli* Opa_CEA_ interaction facilitates mucosal colonization *in vivo*

**Fig 2:**
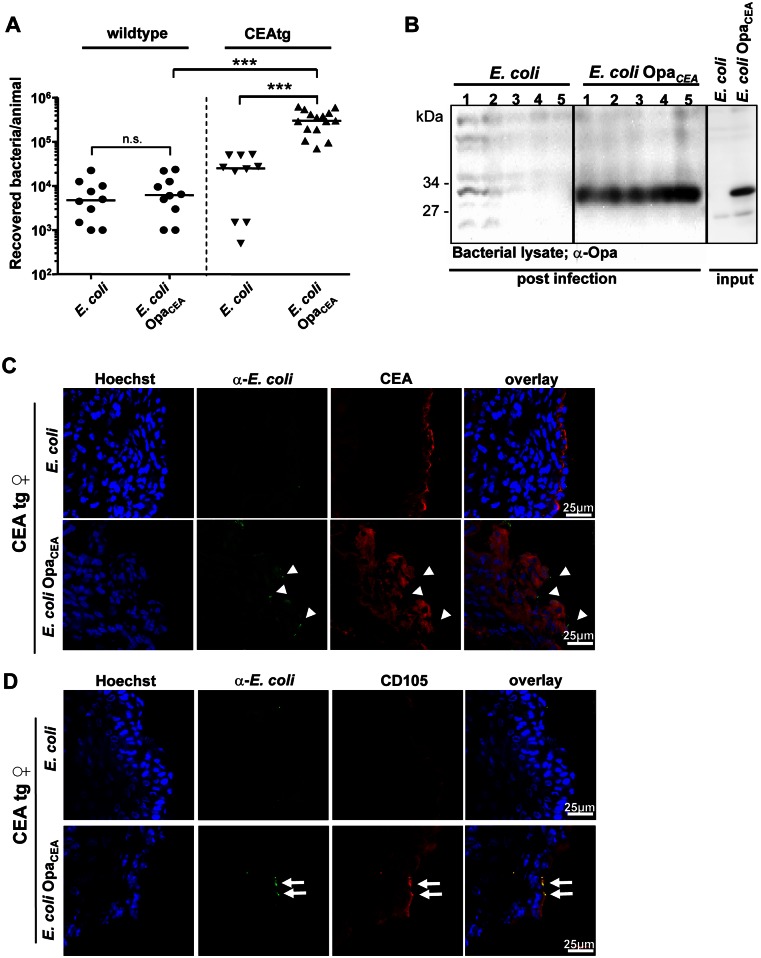
The interaction of *E*. *coli* Opa_CEA_ with CEA facilitates mucosal colonization and leads to CD105 expression in epithelial cells. *(A)* Wild-type (●) or CEAtg (▼) female mice were infected with the indicated bacterial strains and 24 h later, bacteria were re-isolated. Each data point in the graph reflects the number of bacteria re-isolated from an individual animal (n = 10; except for CEAtg animals infected with *E*. *coli* Opa_CEA_, where n = 15). Data were compiled from four independent experiments. The median for each experimental group of animals is indicated by a line; groups were compared by Mann-Whitney U-test and highly significant differences (p<0.001) are indicated by ***. *(B)* Individual re-isolated bacterial colonies from the genital tract of CEAtg mice infected with either *E*. *coli* or *E*. *coli* Opa_CEA_ were plated on LB-ampicillin agar plates (post infection). Five isolates for each strain were analysed by Western blotting with an antibody against Opa protein. As a control, lysates of the *E*. *coli* or *E*. *coli* Opa_CEA_ used for infection (input) were also analysed. *(C)* Genital tracts from CEAtg mice infected for 24 hours with *E*. *coli* or *E*. *coli* Opa_CEA_ were excised, and cryosections were co-stained with antibodies against *E*. *coli* (green) and against CEA (red). Cell nuclei were stained with Hoechst dye (blue). Arrowheads indicate host-associated *E*. *coli*. Pictures are representative for three independent biological replicates. *(D)* Cryosections as in (C) were co-stained with antibodies against *E*. *coli* (green) and a rat monoclonal antibody against murine CD105 (red). Cell nuclei were visualized by Hoechst (blue). CD105 expression on the mucosal surface of CEAtg mice infected with *E*. *coli* Opa_CEA_ is highlighted by small arrows. Pictures are representative for three independent biological replicates.

Because Opa_CEA_-expressing *E*. *coli* was able to enhance integrin activity and suppress cell detachment *in vitro*, we next analyzed bacterial colonization of the urogenital tract. For this purpose, we used either wildtype mice or transgenic mice, expressing human CEA on all mucosal surfaces (CEAtg mice) [14,23]. Accordingly, 8–10 week old female mice were vaginally infected with 1 x 10^6^ bacteria and colonizing bacteria were recovered by urogenital swabs 24 hours later. Only few bacterial colonies of the *E*. *coli* control strain could be isolated from wildtype mice and a slightly elevated (~3-fold) recovery of this non-CEACAM binding strain from CEA-tg mice was observed (Fig 2A). In contrast, more than 30-fold higher numbers of Opa_CEA_ protein-expressing *E*. *coli* were recovered from CEAtg mice than from wildtype mice (Fig 2A). Analysis of re-isolated Opa_CEA_ protein-expressing *E*. *coli* showed that expression of the Opa adhesin was unaltered by *in vivo* growth conditions (Fig 2B). Immunohistochemical staining of tissue sections from the urogenital tract revealed that Opa_CEA_-expressing *E*. *coli* were closely associated with the CEA-positive tissue surface (Fig 2C). In the case of *E*. *coli* control only few bacteria could be detected on the mucosal surface (Fig 2C). Importantly, CD105 was expressed by mucosal epithelial cells of CEAtg mice in contact with Opa_CEA_-protein expressing *E*. *coli*, whereas the *E*. *coli* control strain did not trigger CD105 expression (Fig 2D). Furthermore, *E*. *coli* did not trigger CD105 expression in wildtype mice, irrespective of the Opa protein status of the bacteria (S6 Fig). These data demonstrate that Opa_CEA_-expressing *E*. *coli* are able to associate with CEA-positive epithelial cells and trigger CD105 expression in the urogenital tract *in vivo*. Together, these data suggest that a CEACAM-binding adhesin is not only necessary, but also sufficient to promote colonization of the mucosal surface via stimulating CD105 expression and enhanced integrin activity in superficial epithelial cells.

### Uropathogenic *E*. *coli* AfaE-III engage CEACAMs in a species-specific manner

**Fig 3:**
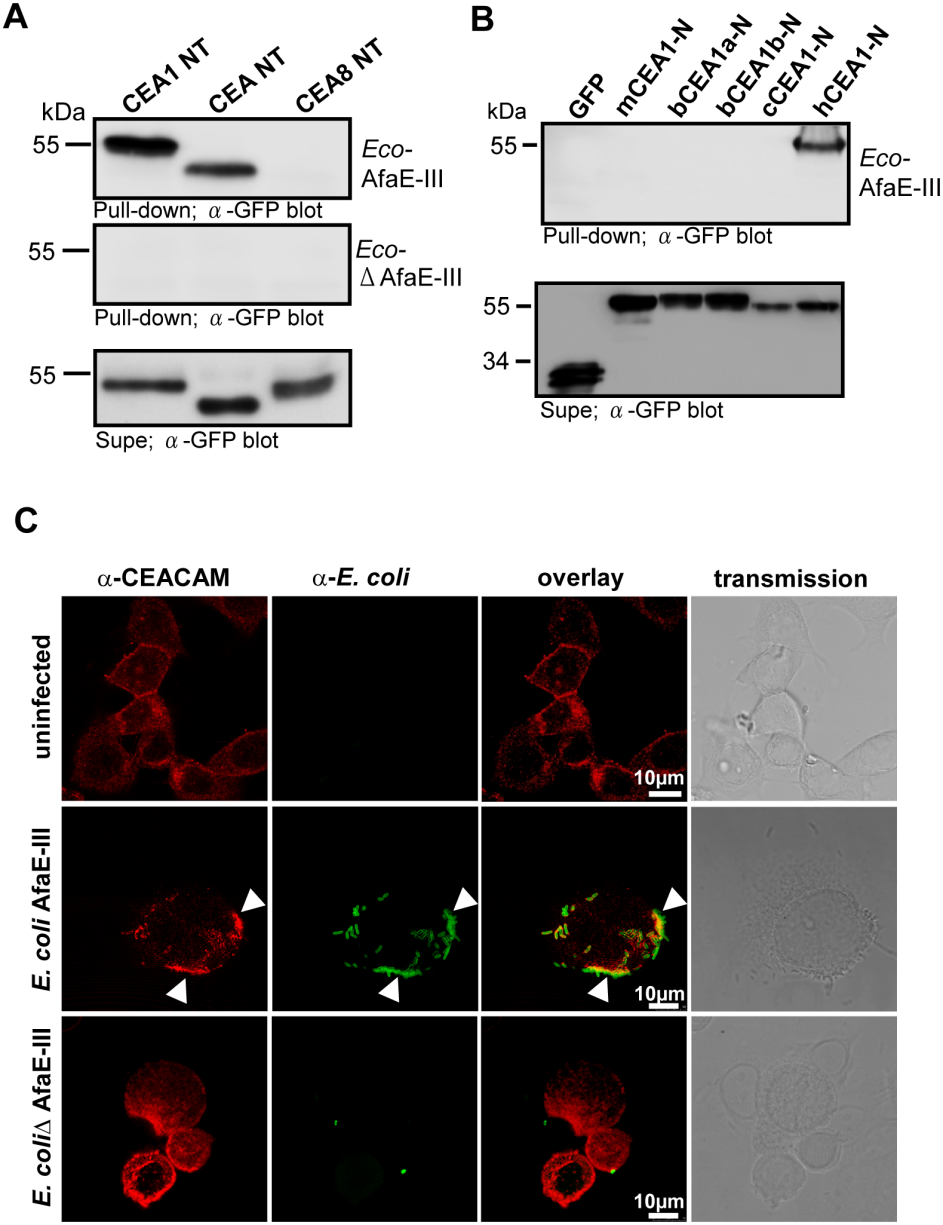
Uropathogenic *E*. *coli* expressing the AfaE-III adhesin selectively bind to the amino terminal domain of human CEACAM family members. *(A)* The indicated soluble CEACAM-GFP fusion proteins (human CEACAM1-NT, CEA-NT, and CEACAM8-NT) were produced in 293 cells and the resulting cell culture supernatants were incubated with *E*. *coli* AfaE-III or with the AfaE-III-deficient strain (*E*. *coli* ΔAfaE-III). After washing, bacteria-associated CEACAM-GFP fusion proteins were detected by Western blotting with a monoclonal anti-GFP antibody (Pull-down; upper two panels). To verify the presence of equal amounts of the CEACAM-GFP fusion proteins, cell culture supernatants were analyzed by Western blotting with anti-GFP antibody (Supe; lowest panel). Shown is a representative experiment out of three independent biological replicates. (*B*) Cell culture supernatants containing the soluble N-terminal domains of murine CEACAM1 (mCEA1), bovine CEACAM1a (bCEA1a), bovine CEACAM1b (bCEA1b), canine CEACAM1 (cCEA1), human CEACAM1 (hCEA1) as GFP-fusion proteins, or GFP alone (GFP) were incubated with *E*. *coli* AfaE-III. After washing, bacteria-associated CEACAM1 was detected as in (A) (Pull-down; upper panel). The presence of CEACAM-GFP fusion proteins was analyzed as in (A) (Supe; lower panel). Shown is a representative experiment out of three independent biological replicates. *(C)* ME-180 cells were infected or not with the indicated bacteria for 2 h, fixed and co-stained with antibodies against endogenous CEACAMs (clone D14HD11; red) and rabbit anti-*E*. *coli* (green). Arrowheads indicate bacteria bound to clustered CEA. Pictures are representative for four independent biological replicates.

Besides the neisserial Opa_CEA_ proteins, additional CEACAM-binding bacterial proteins have been described. For example, uropathogenic *E*. *coli* harbouring the Afa/Dr locus of afimbrial adhesins have also been shown to engage CEACAMs including CEACAM1 and CEA [24]. Therefore, we used the uropathogenic *E*. *coli* (UPEC) strain A30, which expresses the AfaE-III adhesin (*E*. *coli* AfaE-III) encoded within the *afa* gene cluster on a large virulence plasmid [25]. To monitor AfaE-III-dependent events, we cured strain A30 from the virulence plasmid generating the AfaE-III-negative strain *E*. *coli* ΔAfaE-III, which lacks the *afa* gene cluster (S7A Fig). The wildtype UPEC strain and the ΔAfaE-III strain grew with similar growth kinetics (S7B Fig). Clearly, *E*. *coli* AfaE-III was able to associate with human CEACAM1 and CEA in the form of soluble GFP-tagged receptor domains and this property was lost in *E*. *coli* ΔAfaE-III (Fig 3A). Similar to other CEACAM-binding bacteria such as gonococci, *E*. *coli* AfaE-III selectively bound to human CEACAM1, but not to CEACAM1 orthologues from other mammalian species, including mouse, dog and cattle (Fig 3B). Furthermore, *E*. *coli* AfaE-III strongly associated with CEA-expressing ME-180 cells and clustered CEA on the surface of the infected cells, whereas *E*. *coli* ΔAfaE-III hardly attached to the cell surface (Fig 3C). These results suggest that *E*. *coli* pathovars associated with urogenital infections target human CEACAM family members present on epithelial cells of the urogenital tract via their AfaE-III adhesin.

### CEACAM-binding uropathogenic *E*. *coli* AfaE-III promotes integrin activity and epithelial cell adhesion

**Fig 4:**
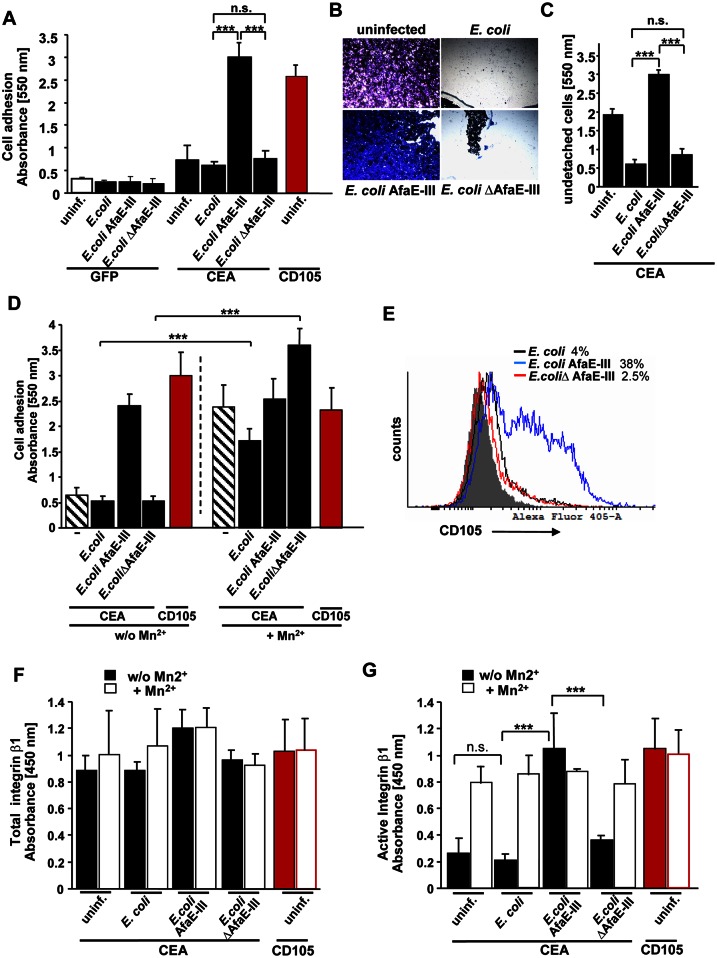
CEA engagement by *E*. *coli* AfaE-III triggers enhanced cell-matrix adhesion via integrin activation. *(A)* 293 cells were transfected with a control vector (GFP), or plasmids encoding CEA or CD105. Cells were left uninfected or infected for 8 h with the indicated bacteria. After infection, cells were used in adhesion assays on collagen. Bars represent means ± S.D. of 8 replicates. Two-tailed student’s t-test; *** p < 0.001, n.s.–not significant. Shown is one representative experiment out of three independent biological replicates *(B)* 293 cells were transfected with CEA and seeded on 25 μg/ml collagen. Confluent layers were left uninfected or infected for 14 h with *E*. *coli*, *E*. *coli* AfaE-III, or *E*. *coli* ΔAfaE-III. Following infection, cells were washed and remaining cells were stained with crystal violet. Representative areas with remaining cells were photographed. Pictures are representative for three independent biological replicates. *(C)* 293 cells were infected and stained as in (B). Staining intensity was determined after dye elution in a spectrophotometer at 550 nm. Bars represent mean ± S.D. of 8 replicates. Two-tailed student’s t-test; *** p < 0.001, n.s.—not significant. Shown is one representative experiment out of three independent biological replicates. *(D)* 293 cells were transfected with plasmids encoding CEA or CD105. Cells were left uninfected or infected for 8 h with the indicated bacteria. After infection, cells were used in adhesion assays on collagen in the absence or presence of 1 mM Mn^2+^. Bars represent means ± S.D. of 8 replicates. Two-tailed student’s t-test; *** p < 0.001, n.s.—not significant. Shown is one representative experiment out of three independent biological replicates. *(E)* CEA-transfected 293 cells were infected for 14 h with the indicated bacteria and analyzed by flow cytometry for CD105 expression. Gray area indicates staining of uninfected cells with an isotype matched control antibody. Shown is one representative experiment out of three independent biological replicates. *(F*, *G)* 293 cells were transfected with plasmids encoding either CEA or CD105. As indicated, transfected cells were infected with bacteria for 14 h or left uninfected. After infection, cells were replated onto collagen-coated culture dishes for 90 min and stimulated or not for 5 min with 1 mM Mn^2+^ before fixation. Fixed samples were either stained with a rat monoclonal integrin β1 antibody (clone AIIB2), which recognizes the integrin β1 extracellular domain irrespective of its conformation (total integrin) (F) or samples were stained with an activation-epitope specific rat monoclonal integrin β1 antibody (clone 9EG7), which recognizes the extended, ligand-bound conformation of integrin β1 (active integrin)(G). Bars represent the mean ± S.D. of 5 replicates. Two-tailed student’s t-test; *** p < 0.001, n.s.—not significant. Shown is one representative experiment out of three independent biological replicates.

To investigate, if a CEACAM-binding pathogenic *E*. *coli* strain is able to modulate the matrix-adhesion of infected epithelial cells, we again employed 293 cells transfected either with a GFP-encoding control vector or a CEA-encoding expression vector. As observed for CEACAM-binding gonococci, *E*. *coli* AfaE-III promoted cell-matrix adhesion in 293 cells expressing CEA, but not in control transfected cells (Fig 4A). Neither the *E*. *coli* control strain nor *E*. *coli* ΔAfaE-III led to enhanced extracellular matrix adhesion of infected cells (Fig 4A). In agreement with the enhanced extracellular matrix adhesion, CEA-expressing 293 cells did not detach upon infection with *E*. *coli* AfaE-III, whereas detachment of cells infected with non-CEACAM-binding *E*. *coli* (*E*. *coli* control strain or *E*. *coli* ΔAfaE-III), could be readily detected under the microscope (Fig 4B and 4C). Treatment of the infected cells with Mn^2+^, a general inducer of integrin activity, increased the matrix adhesion of cells infected with the *E*. *coli* control strain or *E*. *coli* ΔAfaE-III, but did not further enhance collagen binding of cells infected with CEACAM-binding *E*. *coli* AfaE-III (Fig 4D). Moreover, infection of CEA-expressing 293 cells with *E*. *coli* AfaE-III, but not *E*. *coli* ΔAfaE-III, triggered CD105 expression by 293 cells (Fig 4E). These results indicate that *E*. *coli* AfaE-III is able to exploit CEACAMs to modulate host cell adhesion and to counteract bacteria-induced cell detachment via CD105 expression and integrin activation. In line with this idea, infection with *E*. *coli* AfaE-III, but not with but not *E*. *coli* ΔAfaE-III, resulted in enhanced integrin β1 activity in CEA-expressing cells (Fig 4F and 4G). These results demonstrate that CEACAM-binding pathogenic strains of *E*. *coli* can modulate host cell adhesion and integrin activity on human epithelial cells.

### *E*. *coli* AfaE-III colonization of the urogenital tract is facilitated by CEACAM-binding and results in CD105 expression in vivo

**Fig 5:**
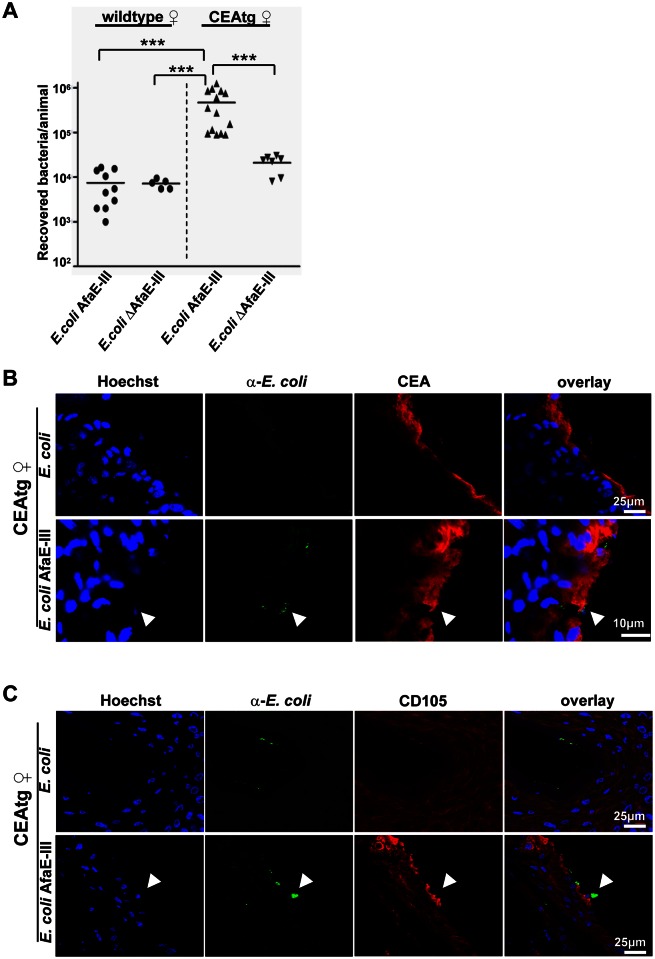
*E*. *coli* AfaE-III shows improved mucosal colonization and induces CD105 expression. *(A)* Wild-type or CEAtg female mice were infected with *E*. *coli* AfaE-III or *E*. *coli* ΔAfaE-III. 24 h later, bacteria were re-isolated. Each data point in the graph reflects the number of bacteria re-isolated from an individual animal (n = 10). Data were compiled from two independent experiments. The median for each experimental group of animals is indicated by a line; numbers of recovered bacteria were compared by Mann-Whitney U-test and highly significant differences (p<0.001) are indicated by ***. *(B*, *C)* Animals were infected as in (A) and genital tracts were excised, fixed and frozen. *(B)* Cryosections of genital tracts were co-stained with rabbit antibodies against *E*. *coli* (green) and a mouse monoclonal antibody against CEA. Cell nuclei were visualized by Hoechst (blue). Numerous *E*. *coli* AfaE-III can be detected in close association with the CEA-positive epithelium (arrowhead), whereas non-CEACAM binding *E*. *coli* are rarely observed. (C) Cryosections were co-stained with rabbit antibodies against *E*. *coli* (green) and a rat monoclonal antibody against murine CD105 (red). Cell nuclei were visualized by Hoechst (blue). Strong local expression of CD105 can be observed on the mucosal surface of CEAtg mice upon association with *E*. *coli* AfaE-III (arrowhead). Pictures in B) and C) are representative for three independent biological replicates.

Because CEACAM-binding by *E*. *coli* AfaE-III results in enhanced integrin activity *in vitro*, we wondered whether these pathogens have an advantage during colonization of the urogenital tract. Therefore, wildtype or CEAtg mice were vaginally infected for 24 h with 10^6^ *E*. *coli* AfaE-III or with *E*. *coli* ΔAfaE-III. 24h after infection, colonization was analyzed by dilution plating. Whereas only low numbers of bacteria were recovered from the urogenital tract of wildtype mice, recovery of *E*. *coli* AfaE-III from CEAtg mice increased more than 80-fold (Fig 5A). In contrast, numbers of non-CEACAM-binding *E*. *coli* ΔAfaE-III were only slightly (~3-fold) elevated in CEAtg mice compared to wildtype mice (Fig 5A). Immunohistochemistry revealed that numerous *E*. *coli* AfaE-III were found associated with the epithelial surface of CEAtg mice, which stained positive for human CEA (Fig 5B). Furthermore, superficial epithelial cells of CEAtg mice infected with *E*. *coli* AfaE-III showed local expression of CD105 (Fig 5C). While occasionally non-CEACAM-binding *E*. *coli* were also detected on the mucosal surface of CEAtg mice, no increase in CD105 expression was evident (Fig 5C). Together, these results indicate that the enhanced colonization of CEAtg mice by *E*. *coli* AfaE-III might be due to modulation of epithelial cell-extracellular matrix adhesion via CD105 expression and integrin activation, which would provide the mechanistic explanation for the observed phenotype.

### CEACAM-binding by *E*. *coli* AfaE-III suppresses epithelial exfoliation in the genital tract

**Fig 6:**
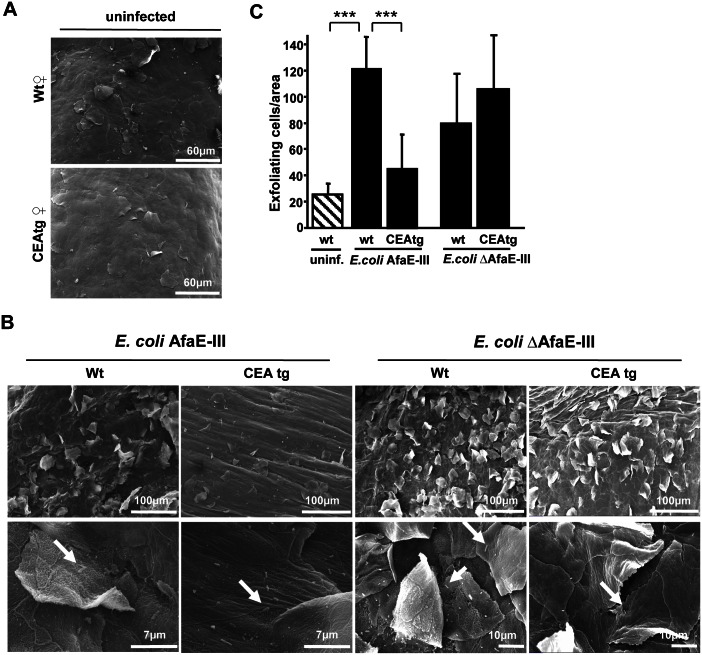
*E*. *coli* AfaE-III suppresses epithelial exfoliation in CEAtg mice. *(A)* Whole mount urogenital tracts of uninfected wild-type or CEAtg female mice were fixed and processed for scanning electron microscopy (SEM). Pictures show the luminal surface of the upper vaginal and cervical regions and are representative for five animals each treatment group. *(B)* Wild-type or CEAtg female mice were infected with *E*. *coli* AfaE-III or *E*. *coli* ΔAfaE-III for 24 h and the genital tracts processed as in (A). SEM pictures (at two different magnifications, as indicated by the scale bars) show the luminal surface of the upper vaginal and cervical regions. Whereas massive epithelial exfoliation is evident in infected wildtype mice and in CEAtg mice infected with *E*. *coli* ΔAfaE-III, a strongly reduced detachment of epithelial cells is observed in CEAtg mice infected with CEACAM-binding *E*. *coli* AfaE-III. Adherent bacteria can be observed at higher magnifications (arrows). Pictures are representative for five animals each treatment group. *(C)* Quantification of exfoliating epithelial cells from samples in (B). Bars represent mean ± S.D. of exfoliating cells from at least n = 26 areas (~0.075 mm^2^) derived from at least five animals each treatment group. Results were compared by Mann-Whitney U-test and highly significant differences (p<0.001) are indicated by ***.

In the case of gonococcal infection, bacteria-triggered CD105 expression on the epithelial surface translates into suppression of host cell exfoliation in vivo [14]. To investigate the level of host cell exfoliation in response to bacterial infection, we used scanning electron microscopy (SEM) of the urogenital tract. In uninfected wildtype and CEAtg animals, the surface of the upper vaginal epithelium showed few detaching superficial cells, indicating low tissue turnover under these conditions (Fig 6A). However, infection of wildtype mice with *E*. *coli* AfaE-III or *E*. *coli* ΔAfaE-III triggered a dramatic increase in exfoliation of superficial epithelial cells (Fig 6B and 6C). In strong contrast, infection with the CEACAM-binding UPEC strain did not result in an increased exfoliation of epithelial cells in CEAtg mice (Fig 6B and 6C). Despite the presence of numerous *E*. *coli* AfaE-III as well as *E*. *coli* ΔAfaE-III on the vaginal epithelium, only the non-CEACAM-binding strain *E*. *coli* ΔAfaE-III led to a strong increase in exfoliation (Fig 6B and 6C). The low number of exfoliating cells in CEAtg mice upon infection with *E*. *coli* AfaE-III was comparable to the uninfected situation indicating that CEACAM-binding by the AfaE-III adhesin is able to completely suppress the exfoliation response (Fig 6B and 6C). Together, these results suggest that CEACAM-binding pathogenic *E*. *coli*, such as *E*. *coli* AfaE-III, can interfere with the detachment of infected epithelial cells in a manner reminiscent of Opa_CEA_-expressing *Neisseria gonorrhoeae*. Suppression of exfoliation clearly is a means to increase the likelihood of a successful and lasting colonization. Therefore, CEACAM-triggered interference with epithelial exfoliation seems to be more common amongst human pathogens than previously appreciated and appears to be an evolutionarily favourable strategy to colonize human mucosal surfaces.

## Discussion

Regulation of epithelial exfoliation is a particularly effective and rapid innate defense mechanism modulating mucosal colonization by microorganisms. However, there is only limited knowledge how pathogens themselves regulate this process and which molecular factors affect cell exfoliation during the course of an infection.

In this study we provide novel insight into the role of bacterial and host determinants, which modulate the exfoliation of epithelial cells. Based on prior observations with *Neisseria gonorrhoeae*, we were able to confirm that engagement of members of the CEACAM family on the mucosal surface of the upper vaginal epithelium results in suppression of exfoliation. Importantly, the observed host cell responses were independent of the bacterial background, in which the CEACAM-binding adhesin was expressed, as both Opa_CEA_ protein-expressing gonococci as well as *E*. *coli* were able to block exfoliation. These results demonstrate that CEACAM engagement is not only necessary, but also sufficient to promote increased host cell-extracellular matrix adhesion and to counteract exfoliation. Interestingly, a pathogenic UTI isolate of *E*. *coli*, which expresses the Dra/AfaE CEACAM-binding adhesin, was able to induce a similar host cell phenotype *in vitro*, characterized by CEACAM-triggered upregulation of CD105, increased integrin activity, and enhanced host cell adhesion to the extracellular matrix. CEACAM engagement allowed these pathogens to blunt epithelial exfoliation leading to enhanced mucosal colonization *in vivo*. Based on these findings we propose that CEACAM-binding adhesins have independently evolved in multiple gram-negative bacterial pathogens, including pathogenic *Neisseriae*, *E*. *coli* pathovars, *Haemophilus influenzae* and *Moraxella catarrhalis*, as a means to facilitate the initial, species-specific contact with the mucosa of an appropriate host organism and to counteract the detachment of superficial cells.

One of the best studied examples of bacteria-induced exfoliation is taking place in the bladder, where incoming bacteria trigger massive shedding of the superficial umbrella cells, a specialized cell type covering the luminal surface of the bladder urothelium [10,26]. Indeed, while in some organs the epithelium regenerates constantly, the mammalian urinary bladder can shift from a physiological mode of slow tissue turnover to a highly proliferative status as a result of epithelial injury [27,28]. UPEC strains that infect the bladder epithelium are characterized by the possession of specific adhesin gene clusters, such as the operons encoding for the type 1 pilus, the P pilus, or the Afa/Dr family of adhesins, as well as the secretion of toxins such as α-hemolysin (HlyA) [29,30]. In particular, the FimH adhesin-mediated contact of UPEC with bladder cells has been shown to induce pronounced exfoliation and tissue renewal [10]. Detachment of the large superficial urothelial cells, which seems to be accompanied by apoptosis, then affords the pathogen access to deeper strata of the bladder epithelium, where some bacteria invade, multiply, and persist in undifferentiated epithelial cells [31,32]. Accordingly, a small fraction of FimH-expressing UPEC seems to profit from exfoliation, as increased urothelial stem and early progenitor cell proliferation provide an expanded protective niche for UPEC in the bladder [27]. In line with the idea that in some instances the bacteria might benefit from the host tissue response, Dhakal and Mulvey recently identified the pore-forming toxin α-hemolysin (HlyA) as the bacterial effector that induces exfoliation upon infection with UPEC [33]. At sublethal concentrations, HlyA activates host cell proteases resulting in breakdown of integrin-associated proteins such as paxillin, thereby weakening cell-matrix attachment.

Interestingly, the finding by Dhakal and Mulvey that a secreted factor promotes cell detachment, also demonstrates that host cell contact is not a pre-requisite for the induction of epithelial exfoliation. This is also reflected in our current and prior studies, where non-adherent bacteria were able to trigger this process in vitro and in vivo [14, 16]. Clearly, the non-pathogenic *E*. *coli* or non-opaque gonococci used in these studies do not secrete toxins, pointing to the existence of additional soluble triggers for host cell detachment. It has been speculated that conserved bacterial factors such as LPS could initiate this host response [8]. However, infection of TLR-4-deficient mice with *E*. *coli* also resulted in a strong increase in exfoliation, suggesting that LPS, or more precisely LPS sensing by TLR-4, is not required to trigger exfoliation (S8 Fig). Therefore, future efforts should be directed towards identifying the relevant (and most likely conserved) bacterial feature(s), which initiate epithelial exfoliation.

Amongst UTI isolates of *E*. *coli*, type 1 pilus or P pilus expression in combination with the secretion of HlyA prevails, which is in line with the idea that these pathotypes profit from epithelial exfoliation [13]. However, a further pathotype of UTI has been described (pathotype V), which lacks HlyA secretion and expresses Afa/Dr-related adhesins [30]. Indeed, non-hemolytic, Afa/Dr-possessing *E*. *coli* are found in less than 4% of fecal isolates, but make up almost 10% of UTI isolates suggesting a prominent enrichment of strains with this genetic makeup during urogenital colonization [30]. Even higher rates of Afa/Dr-expressing strains have been reported from cystitis cases in children and pyelonephritis cases of pregnant women [34,35]. Our results with a non-hemolytic, AfaE-III-expressing strain now demonstrate that this pathotype can engage human CEACAMs on the surface of the urogenital mucosa to suppress exfoliation. Though our model relies on infection of the upper vaginal epithelium in mice, rather than the bladder epithelium, pathogenic *E*. *coli* can also infect the human genital tract mucosa of both male and female and can be acquired similar to gonococci by sexual transmission [36]. Therefore, our results indicate that amongst isolates some strains may, in contrast to HlyA-expressing strains, be able to suppress exfoliation via the possession of CEACAM-binding Afa/Dr adhesins.

Clearly, CEACAM-binding is only found in a subfamily of Afa/Dr adhesins expressed by *E*. *coli*, the so-called Afa/Dr-I family, including Dr, F1845 and AfaE-III adhesins [24,37]. The Afa/Dr adhesins belong to the large group of chaperone/usher (CU) type adhesins, which can be located on fimbriae or which can occur in the form of an afimbrial surface sheath (such as in the case of Afa adhesins) [38,39]. Similar to other CU systems, the Afa adhesins are part of a small gene cluster (*afaA*–*afaF*), which, besides the adhesin, encodes the accessory proteins required for surface expression of the adhesin proper [38]. The *afa* gene clusters comprise genes of transcriptional regulators (*afaA*, *afaF*), a periplasmic chaperone (*afaB*), and an integral outer-membrane protein (*afaC*), which serves as the assembly platform, the so-called usher, for the surface display of the adhesin [40]. In the case of the *afa-3* gene cluster, there are two genes (*afaD* and *afaE*), which encode proteins with adhesive and invasive properties towards mammalian cells [41–44]. Isolated, recombinant AfaE-III derived from the *afa-3* operon of the UPEC strain A30 has been shown to bind to CD55 as well as members of the CEACAM family including CEACAM1, CEA, and CEACAM6 [37]. However, AfaE orthologues from other pathogenic *E*. *coli* strains, such as AfaE-I from strain KS52, seemingly lack CEACAM binding [37]. For AfaE-III, the CEACAM binding interface of the adhesin has been structurally defined [45]. Though the six amino acids of AfaE-III engaged in the binding interface with CEA seem to be largely conserved in the CEACAM-binding Dr protein from strain IH11128, the CEACAM-binding adhesin F1845 encoded by the *daaE* gene from strain C1845 does not share a single identical amino acid at the corresponding positions [37,46,47].

The lack of a defined CEACAM-binding motif currently prohibits the use of the vast sequence information about *E*. *coli* adhesins to predict possible binding interactions with human CEACAMs. Furthermore, a comprehensive functional analysis of CEACAM-binding properties among UTI-causing *E*. *coli* isolates and a correlation with the clinical manifestations is currently lacking. It is therefore interesting to note, that a recent survey by Qin et al. found a strong association between the presence of the *afa* gene cluster and recurrent infections of the lower urogenital tract, which were characterized by a lack of systemic symptoms [48]. In contrast, the *afa* operon was not detected in strains isolated from acute pyleonephritis or cystitis patients [48]. These findings are in line with the idea that some pathogenic strains of *E*. *coli*, e.g. *afa-3* harboring strains, by the help of CEACAM-binding adhesins, are able to sustain long-term accommodation in the urogenital tract without causing systemic pathology. Interestingly, also many asymptomatic bacteriuria (ABU) *E*. *coli* isolates are hlyA-negative and carry *afa* gene clusters [49]. Based on our previous study, we have performed an additional extended PCR screening of 126 ABU isolates from various sources and find that 9.3% of these isolates encode *afa/dra* genes. These ABU strains can colonize the urinary tract with high bacterial numbers for many weeks or months without provoking an overt innate immune response [50]. Accordingly, suppression of exfoliation via Afa/Dr binding to CEACAM may also promote the stable asymptomatic colonization of the bladder by ABU strains. Given the HlyA-triggered induction of exfoliation versus Afa/Dr-mediated suppression of exfoliation, it can be envisioned that the presence of the respective virulence factors might dictate the distinct behaviour of these strains and the clinical outcome.

Importantly, the majority of CEACAM-binding bacteria characterized to date does not colonize the urogenital tract, but rather inhabits the nasopharynx, such as *Neisseria meningitidis*, *Moraxella catarrhalis* and *Haemophilus influenzae* [51–53]. In each case, the bacteria employ structurally unrelated adhesins to contact CEACAMs on the luminal surface of the host tissue, where CEACAM-binding adhesins, including AfaE-III, bind to the protein part and not the glycan part of the receptor [24, 45,54–56]. It is easily conceivable that all these human-restricted pathogens exploit CEACAMs for securing successful colonization of their sole natural host. Indeed, recent experiments with model organisms have demonstrated that adhesin-CEACAM interactions contribute to improved recovery of these bacteria after experimental infection of the nasopharyngeal mucosa [19, 57]. Our in vivo experiments with CEAtg-mice indicate that the recovery of non-CEACAM binding *E*. *coli* from CEA-transgenic animals is also slightly, but consistently elevated compared to wildtype animals. It could be speculated that by expressing human CEA in mice, an additional glycoprotein is added to the murine mucosa that could allow additional low affinity, glycan-based interactions. Though such weak interactions might not be apparent in in vitro binding assays with soluble CEA-domains, they could contribute to a slightly improved colonization in vivo. Clearly, there is a strong further increase in colonization of the urogenital tract by CEACAM-binding *E*. *coli* and this correlates with the ability of these strains to suppress exfoliation in vivo, an effect not observed for non-CEACAM-binding bacteria. This suggests that potential low affinity, glycan-mediated binding interactions are not sufficient to mediate suppression of epithelial exfoliation. In contrast to the situation in the urogenital tract, it is currently unknown, if the improved colonization of the nasal cavity of CEACAM1 transgenic mice observed for *N*. *meningitidis* or the CEACAM-dependent colonization of chinchillas by *Haemophilus influenzae* is linked to a suppression of exfoliation. However, at least in stratified regions of the nasopharyngeal epithelium similar processes might occur as observed for AfaE-III-expressing *E*. *coli* or Opa_CEA_ protein expressing *N*. *gonorrhoeae* in the urogenital tract. Together, a detailed understanding of the molecular processes initiating the exfoliation of epithelial cells and the host-tailored countermeasures by successful pathogens holds the promise to provide novel avenues to interfere with or to protect from bacterial colonization right from the start of the bacterial host cell encounter.

## Materials and Methods

### Bacteria and infection

The *E*. *coli* and *E*. *coli* Opa_CEA_ strains were derived from DH5α and have been described previously [22]. The heterologous expression of Opa proteins in *E*. *coli* allows these strains to interact with human CEACAMs in a manner analogous to Opa protein expressing gonococci and meningococci [18]. The opaque phenotype was regularly controlled by Western blotting of bacterial lysates with the monoclonal anti-Opa protein antibody (clone 4B12/C11). The uropathogenic *E*. *coli* harbouring the *afa-3* gene cluster (strain A30; isolated from a cystitis patient [25,58] is a non-hemolytic, serotype O75 strain expressing the CEACAM-binding AfaE-III adhesin (*E*. *coli* AfaE-III). All *E*. *coli* strains were grown on LB agar plates with or without adequate antibiotics and cultured at 37°C. To select for *afa*-deficient variants of *E*. *coli* AfaE-III, bacteria were sequentially cultured overnight at 37°C, then for 24 h at 40°C, at 42°C, and at 45°C. Serial dilutions of the final culture were plated on LB agar and individual colonies were tested by colony-PCR for the presence of the *afa* locus (primer pair afa-f: 5’-ggcagagggccggcaacaggc-3’; afa-r: 5’-cccgtaacgcgccagcatctc-3‘) and the K5 capsule determinant (K5-f: 5‘-cagtatcagcaatcgttctgta-3‘ / kpsII-r: 5’-catccagacgataagcatgagca-3’) as described [59].

In addition, we have attempted to generate a complemented strain, but have not succeeded in re-expressing the *afa*-gene cluster of *E*. *coli* strain A30, despite subcloning into low-copy or inducible vectors. Sequencing of subcloned gene clusters in multiple independent clones revealed that all the tested subclones carried point mutations resulting in premature stop codons or frame shifts within the coding regions of the *afa*-gene cluster, indicating that the maintenance of heterologous fimbrial gene clusters in multiple copies in *E*. *coli* laboratory strains may select for subcloned PCR products with accumulated mutations preventing expression. For determining growth curves of *E*. *coli* strains, LB broth cultures were initiated and grown at 37°C for 4 h. These cultures were used as 1:25 inoculation into 100 ml LB medium at 37°C. Absorbance at 600nm was recorded every 60 min over a course of 10–25 h using a Libra S4 spectrophotometer (Biochrom, Cambridge, UK). For infection, bacteria were suspended in DMEM, the optical density of the suspension at 600 nm (OD_600_) was used to estimate the number of bacteria according to a standard curve, and the bacteria were added to the cells at the indicated multiplicity of infection (MOI).

*Neisseria gonorrhoeae* strains used in this study were described previously and were derived from strain MS11 [16]. The gonococci were either non-piliated and expressed a CEACAM-binding Opa protein (Ngo Opa_CEA_; strain N309), were non-piliated and expressed a heparansulphate proteoglycan-binding Opa protein (Ngo Opa_HSPG_; strain N303) [22], or the bacteria did not express an Opa protein (non-opaque phenotype), but expressed pili to bind to human cells (Ngo P+, strain N280, a non-opaque derivative of MS11-F3 [60]). The opacity and the piliation status of the used bacteria were regularly monitored by colony morphology as well as Western Blotting with monoclonal anti-Opa antibodies. Gonococci were grown on GC agar plates (Difco BRL, Paisley, UK) supplemented with vitamins, chloramphenicol (10 μg/ml) and erythromycin (7 μg/ml) at 37°C, 5% CO_2_ and subcultured daily.

### Cell culture and plasmid transfection

The human cervix carcinoma cell line ME-180 (ATCC, Rockville, MD) and the embryonic kidney cell line 293T (293 cells; ACC-635, DSMZ, Braunschweig, Germany) were cultured in DMEM containing 10% calf serum (293 cells) or DMEM containing 10% FCS (ME-180 cells) at 37°C in 5% CO_2_ and subcultured every second to third day. 293 cells were transfected by calciumphosphate co-precipitation using a total of 5 μg of plasmid DNA for each 10 cm culture dish and employed in experiments 2 days after transfection. In some cases, 293 cells were serum-starved in DMEM containing 0.5% CS. The human vaginal epithelial cell (hVEC) line MS74 was obtained from A.J. Schaeffer (Feinberg School of Medicine, Northwestern University, Chicago, IL), cultured on gelatine-coated dishes in DMEM containing 10% fetal calf serum (FCS), and subcultured every third day.

### Recombinant DNA

The expression plasmids used in this study included the commercially available vectors pcDNA3.1 Hygro (pcDNA; Invitrogen, Karlsruhe, Germany), pEGFP-N1, pLPS-3’-EGFP and pDsRed2-C1 (Clontech, Palo Alto, CA). Plasmid pcDNA3.1 CEA (pcDNA-CEA) was described previously [61]. The expression plasmid pcDNA3.1 CEACAM1-4L-HA (pcDNA-CEACAM1) was constructed by PCR amplification of the human CEACAM1-4L cDNA (generous gift of W. Zimmermann, LMU München, Germany) with primers CEACAM1-HA-sense (5’-GGGAAGCTTGCCATGGGGCACCTCTCAGCCCCACTTCAC-3’) and CEACAM1-HA-anti (5’-GGGGACGTCATAGGGATACTGCTTTTTTACTTCTGAATAAATTATTTCTG-3’) and was cloned into the HindIII-AatII—digested plasmid pBluescript CEACAM3-HA [62] before further subcloning via HindIII-NotI into pcDNA3.1 Hygro (Invitrogen). The vector pLPS-3'-RFP2 was constructed by PCR amplifying the RFP2-coding sequence from vector pDsRed2-C1 with primers RFP2-AgeI-sense 5’-ATAACCGGTCGCCTCCTCCGAGAACGTCATCACC-3’ and RFP2-NotI-anti 5’-ATAGCGGCCGCTTACAGGAACAGGTGGTGGCGGCC-3’ and subcloning into the AgeI/NotI sites of pLPS-3’-EGFP resulting in the exchange of the GFP by the DsRed2 coding sequence. The human CD105 cDNA was transferred by Cre-mediated recombination from pDNR-dual CD105 [16] into pLPS-3'-RFP2 resulting in RFP2 fused to the carboxy-terminus of full-length CD105.

GFP-fusion proteins of the human CEACAM1, CEA, and CEACAM8 amino-terminal IgV-like domains as well as the corresponding constructs encoding the amino-terminal IgV-like domains of CEACAM1 orthologues from dog (cCEACAM1), the two CEACAM1 alleles from cattle (bCEACAM1a; bCEACAMb), and from mouse (mCEACAM1) have been constructed and employed previously [20,63].

### Production of soluble CEACAM N-terminal domains and binding to bacteria

293 cells were transfected with 5 μg plasmid DNA encoding secreted GFP-fusion proteins of the N-terminal domains of human or mammalian CEACAM family members. After 24 h, the medium of the transfected 293 cell cultures was replaced by serum-reduced OptiMEM (Life Technologies, Darmstadt, Germany). Two days later the culture supernatants (supe) containing the secreted fusion proteins were collected, centrifuged for 15 min at 5000 rpm and either stored at -20°C or used immediately for bacterial pull-down experiments. The GFP-derived fluorescence was analysed using a Varioskan Flash reader (Thermo Scientific) to adjust equal amounts of the secreted fusion proteins. For pull-down experiments, indicated bacteria were suspended in PBS and binding to the indicated receptor protein contained in cell culture supernatants was determined essentially as described [63].

### Immunofluorescence staining, cell lysis, Western blotting, and antibodies used

Immunofluorescence staining, cell lysis and Western blotting were performed as described previously [62] using mAbs against CEACAMs (clone D14HD11) against CEACAM1 (clone GM-8G5), against CEACAM6 (clone 9A6) (all from Aldevron, Freiburg, Germany), against CEA (clone COL-1; Zymed, San Francisco, CA), against green fluorescent protein (GFP; clone JL-8; BD Biosciences), against *E*. *coli* LPS (AbD Serotec, Oxford, UK), or against murine CD105 (clone MJ7/18; Southern-Biotech, Birmingham, USA). Mouse monoclonal antibodies against human CD105 (clone P4A4; provided by Developmental Studies Hybridoma Bank (DSHB), University of Iowa) or against Opa proteins (clone 4B12/C11; generous gift of Marc Achtman, University of Warwick, UK) as well as rat monoclonal anti-integrin β1 (clone 9EG7; provided by D. Vestweber (MPI for Molecular Medicine, Münster, Germany)) and rat monoclonal anti-integrin β1 (clone AIIB2; DSHB) were purified from hybridoma culture supernatants. Further, rabbit polyclonal antisera raised against paraformaldehyde-fixed *N*. *gonorrhoeae* MS11 (IG-511) was custom produced by Immunoglobe (Himmelstadt, Germany). All secondary antibodies were from Jackson Immuno-Research (West Grove, PA).

### Electron microscopy of isolated cells

For scanning electron microscopy, ME-180 cells were seeded at 2.5 x 10^5^ cells/well in 24-well plates on acid-washed glass coverslips coated with 25 μg/ml collagen and grown to confluency. Medium was replaced with DMEM, 0.5% FCS for 8 h. Then cells were infected for 14 h at a MOI of 20 or left uninfected. Samples were fixed in situ for at least 1 h at 4°C. The samples were washed and dehydrated in a graded series of aceton on ice. After critical point drying samples were sputter-coated with 5 nm gold-palladium in a BAL-TEC SCD 030 and examined at 15 kV accelerating voltage in a Philipps 505 scanning electron microscope using the secondary electron detector. Images were digitally recorded with a DISS 5 system (remX GmbH, Bruchsal, Germany) and processed in Adobe Photoshop 6.

### Animal husbandry and breeding

C57BL/6J mice transgenic for human CEA (CEAtg mice) have been described before [14,23]. Wildtype C57BL/6J mice (originally obtained from Elevage Janvier, Le Genest Saint Isle, France) and CEAtg mice as well as TLR4+/+(HeN) and TLR-/- (HeJ) female mice were maintained under specified pathogen-free conditions under a 12-h light cycle in the animal facility of University of Konstanz in accordance with the institutional guidelines. The CEAtg mice were kept heterozygous for the transgene by crossing male CEAtg mice with female WT mice. Offspring of these crosses (age 3–4 weeks old) was genotyped by PCR.

### Ethics statement

Experiments involving animals were performed in accordance with the German Law for the Protection of Animal Welfare (Tierschutzgesetz). The animal care and use protocol, including the protocol of experimental vaginal infection of female mice, was approved by the appropriate state ethics committee and state authorities regulating animal experiments (Regierungspräsidium Freiburg, Germany) under the permit file numbers G-10/108 and G-15/43.

### *In vivo* infection of CEAtg and wildtype mice with *E*. *coli* strains

Experimental vaginal infection of female mice with *E*. *coli* strains was performed as previously described for *N*. *gonorrhoeae* [14]. Briefly, CEAtg and wildtype mice were subcutaneously injected with 17-β-estradiol 4 days prior to infection. The drinking water was supplemented with ampicillin (1 mg/ml) to reduce the overgrowth of commensal bacteria during hormone treatment. Ampicillin containing water was changed to normal drinking water 1 day before infection. Mice were inoculated intravaginally with 10^6^ CFU of the different *E*. *coli* strains suspended in 20 μl of PBS. 24h later, the mucosa-associated bacteria were re-isolated by cotton swaps. Serial dilutions of re-isolated bacteria were plated on LB agar (*E*. *coli* AfaE-III) or LB agar containing ampicillin (*E*. *coli* Opa_CEA_ and *E*. *coli*). In order to analyse the Opa protein profile of re-isolated bacteria, single colonies were expanded on agar plates, lysed and analysed by Western blotting using Opa specific antibodies.

### Immunohistochemistry of tissue samples

The genital tract of infected animals was excised and the longitudinally opened vaginal and uteral tissue was mounted and analysed by scanning electron microscopy essentially as described previously [14]. For immunohistochemistry, tissue samples were immediately fixed with 4% paraformaldehyde for at least 24 h and transferred to 10% sucrose, 0.05% cacodylic acid for 1 h at 4°C. Next, samples were transferred to 20% sucrose for 1 h and then into 30% sucrose at 4°C over night. Organs were mounted in embedding medium (Cryo-M-Bed; Bright Instrument, Huntingdon, UK) and frozen at -20°C. 10 μm thick sections were cut at -20°C using a cryostat (Vacutom HM500, Microm, Germany). Sections were incubated with a mouse monoclonal antibody against CEA (clone COL-1; dilution 1:200) or a rat monoclonal antibody against murine CD105 (clone MJ7/18; dilution 1:1200) together with a polyclonal rabbit antibody against *N*. *gonorrhoeae* MS11 (dilution 1:100) or a polyclonal rabbit antibody against *E*.*coli* (dilution 1:200). Detection of the primary antibodies was accomplished by incubation with a combination of Cy5-conjugated goat-anti-rabbit antibody (1:250) and rhodamine-conjugated goat-anti-rat antibody (1:250; in the case of CD105 detection) or Cy3-conjugated goat-anti-mouse antibody (1:250; in the case of CEA detection). Cell nuclei were visualized by the addition of Hoechst 33342 (1:30,000; Life Technologies, Darmstadt, Germany) in the final staining step. Samples were analysed with a TCS SP5 confocal laser scanning microscope (Leica, Mannheim, Germany). Images were digitally processed with Photoshop CS (Adobe Systems, Mountain View, CA) and merged to yield pseudo-coloured images.

### Analysis of receptor expression by flow cytometry

293 cells were transfected with pcDNA3.1 Hygro (pcDNA), pcDNA-CEA, or pLPS-3’-RFP2-CD105. Cells were infected or not with *E*. *coli*, *E*. *coli* Opa_CEA_, *E*. *coli* AfaE-III, or gonococci for 14 h. After infection, cells were stained with monoclonal antibodies against CEA (clone COL-1) or against human CD105 (clone P4A4) for 1 h at 4°C. Following washing, samples were stained with a Cy2-conjugated goat-anti-mouse antibody for 30 min, 4°C. ME180 cells and hVEC cells were analysed for the presence of endogenous CEACAMs using mouse monoclonal antibodies specific for CEACAM1 (clone GM-8G5), CEACAM6 (clone 9A6), or CEA (clone COL-1). Stained samples were analysed for Cy2-derived fluorescence by flow cytometry on an LSRII (BD Biosciences) using FACS Diva software.

### Cell adhesion, cell detachment and integrin activity assays

Cell adhesion and cell detachment assays were performed essentially as described [14,16]. Briefly, the wells of 96-well plates were coated with PBS containing collagen type 1 from calf skin (ICN Biomedicals, Irvine, CA) for 24 hours at 4°C. 293 cells were transfected with pEGFP-N1 (GFP), CEA or pLPS3’-CD105 (CD105). After serum starvation, cells were infected or not with the indicated bacterial strains at a MOI of 30 for 8 hours. Following infection, the cells were detached and kept in suspension medium (DMEM, 0.2% BSA) with or without 1 mM Mn^2+^ (1 h at 37C°), and then replated at 4 x 10^4^ cells/well onto collagen-coated wells. Cells were allowed to adhere for 90 min in the presence or absence of 1 mM Mn^2+^ at 37°C, before non-adherent cells were removed by gentle washing with PBS. For the Mn^2+^-treated samples, the washing buffer also contained 1 mM Mn^2+^. Adherent cells were fixed and stained for 60 min with 0.1% crystal violet in 0.1 M borate, pH 9. After washing and drying, the crystal violet was eluted in 10 mM acetic acid and the staining intensity was measured at 550 nm with a Varioskan Flash (Thermo Fisher Scientific Oy Microplate Instrumentation (Vantaa, Finland).

For measuring integrin activity, cells were serum-starved over-night, before they were infected or not with the indicated bacteria at an MOI of 30 for 8 h. Following infection, cells were detached by limited trypsin/EDTA digestion that was stopped by addition of soybean trypsin inhibitor (0.5 mg/ml in DMEM). Detached cells were kept in suspension medium (DMEM, 0.2% BSA) for 1 h at 37°C, and then replated at 5 x 10^4^ cells/well into wells coated with collagen type 1. After 75 min, wells were treated or not with 1 mM MnCl_2_, incubated for 5 min and transferred to ice. Cells were fixed with 4% paraformaldehyde in PBS for at least 30 min, washed with PBS and permeabilized with Triton X-100 (0.1% in PBS) for 15 min. Cells were washed with PBS and blocked with 2% BSA in PBS (blocking buffer) for 20 min, before incubation for 1 h with rat monoclonal antibodies against active integrin (clone 9EG7; dilution 1:600) or against total integrin (clone AIIB2; dilution 1:750) in blocking buffer as described [14]. After washing and incubation with ProteinA/G-HRP (1:250), 100 μl/well of substrate solution (substrate solution was prepared by mixing 10 ml of 2.4 mg/ml tetramethylbenzidine in 10% acetone, 90% ethanol with 0.5 ml of 30 mM potassium citrate, pH 4.1) were added. The enzymatic colour reaction was stopped using 2 M H_2_SO_4_ (100 μl/well) and the absorbance was determined at 450 nm in a Varioskan Flash (Thermo Scientific) microplate reader.

### Statistical analysis

For cell adhesion, cell detachment, and integrin activity assays, values were analysed for normal distribution and mean values were compared by two-tailed unpaired t-test. For in vivo infection assays, including enumeration of exfoliating cells, differences between samples were assessed using the Mann-Whitney U-test. Differences between samples with p<0.001 are indicated by ***.

## Supporting Information

S1 Fig *E*. *coli* Opa_CEA_ expresses the gonococcal Opa_CEA_ adhesin and grows comparable to the *E*. *coli* control strain. *(A)* Lysates of *E*. *coli*, *E*. *coli* Opa_CEA_, and Opa_CEA_-expressing gonococci (Ngo Opa_CEA_) were analysed by Western blotting with a monoclonal anti-Opa antibody. *(B)* Growth of *E*. *coli* and *E*. *coli* Opa_CEA_ in cell culture medium was monitored using optical density readings at 600 nm (OD600) every 60 min. Data plotted are the means of triplicate cultures. *(C)* Growth of *E*. *coli* Opa_CEA_ in the presence or absence of IPTG was monitored as in (B). Data plotted are the means of triplicate cultures.
(PDF)

S2 Fig *E*. *coli* Opa triggers recruitment of CEACAMs. *(A)* ME-180 cells were analysed for endogenous CEACAMs by flow cytometry using monoclonal mAbs GM-8G5, recognizing CEACAM1 (red line), 9A6, recognizing CEACAM6 (red line), or COL-1, recognizing CEA (red line). Gray areas indicate staining of cells with isotype-matched control antibodies. *(B)* ME-180 cells were seeded on glasscover slips, infected or not with the indicated bacteria for 2 h with an MOI of 20, fixed and stained with antibodies against endogenous CEACAMs using clone D14HD11 (red) and rabbit -*E*. *coli* (green) or -*N*. *gonorrhoeae* (green). Bacteria bound to the CEA-positive cells are indicated by arrowheads.
(PDF)

S3 Fig *E*. *coli* Opa_CEA_ suppresses detachment of primary vaginal epithelial cells. *(A)* Human vaginal epithelial cells (hVECs) cells were seeded in 24-well plates coated with 25 μg/ml collagen. Confluent layers were left uninfected or infected for 14 h with *E*. *coli*; *E*. *coli* Opa_CEA_; piliated, non-opaque *N*. *gonorrhoeae* (Ngo P+); non-piliated gonococci expressing a heparansulphate proteoglycan-binding Opa protein (Ngo Opa_HSPG_); or non-piliated gonococci expressing a CEACAM-binding Opa protein (Ngo Opa_CEA_). Following infection, cells were washed and remaining cells were stained with crystal violet. Representative areas with remaining cells were photographed. *(B)* hVEC cells were infected and stained as in (A). Staining intensity of undetached cells was determined after dye elution in a spectrophotometer at 550 nm. Bars represent mean ± S.D. of 6 wells. *(C)* hVECs were analysed for CEACAM expression by flow cytometry using a mouse monoclonal anti-CEACAM antibody (clone D14HD11; red line). Gray area indicates staining of hVECs with isotype-matched control antibody.
(PDF)

S4 Fig CEA binding by *E*. *coli* is accompanied by increased cell-matrix adhesion and upregulation of CD105. *(A)* 293 cells were transiently transfected with an empty control plasmid or a CEA-encoding plasmid and analysed by flow cytometry. About ~40% of the cell population showed CEA surface expression after transfection as detected by a monoclonal CEACAM antibody. Gray area indicates staining of CEA-transfected cells with an isotype matched control antibody. *(B)* 293 cells were transfected with the empty vector control (pcDNA) or plasmids encoding CEA or CD105. Cells were either left uninfected or infected for 8 h with the indicated bacteria. Then, cells were used in adhesion assays on collagen. Bars represent means ± SD of eight samples. Two-tailed student’s t-test; *** p < 0.001. *(C)* 293T cells transfected with a CEA-encoding plasmid were either left uninfected or infected for 14 h with *E*. *coli*, *E*. *coli* Opa_CEA_, or Ngo Opa_CEA_ and analyzed by flow cytometry with a monoclonal anti-human CD105 antibody. Gray area indicates staining of uninfected cells.
(PDF)

S5 Fig *E*. *coli* Opa_CEA_ trigger enhanced integrin activity via CEACAM stimulation. *(A*, *B)* 293 cells were transiently transfected with plasmids encoding either CEA or CD105. Transfected cells were infected with indicated bacteria for 14 h or left uninfected. Next, cells were replated onto collagen-coated culture dishes for 90 min and stimulated or not for 5 min with 1 mM Mn^2+^ before fixation. Fixed samples were either stained with a rat monoclonal integrin 1 antibody (clone AIIB2), which recognizes the integrin 1 extracellular domain irrespective of its conformation (total integrin 1) (A) or samples were stained with an activation-epitope specific rat monoclonal integrin 1 antibody (clone 9EG7), which recognizes the extended, ligand-bound conformation of integrin 1 (active integrin 1) (B). Bars represent the mean ± s.d. of 5 wells of a representative experiment repeated twice with similar results. *(C*, *D)* 293 cells were transiently transfected with the empty control vector (pcDNA) or a plasmid encoding CD105 and infected as indicated. Total integrin 1 and active integrin 1 (clone 9EG7) were detected as in (A, B). Bars represent the mean ± s.d. of 5 wells of a representative experiment repeated twice with similar results. Two-tailed student’s t-test; *** p < 0.001, n.s.—not significant.
(PDF)

S6 Fig *E*. *coli* Opa_CEA_ does not induce CD105 expression in wildtype animals. Genital tracts from wild-type mice infected for 24 hours with *E*. *coli* or *E*. *coli* Opa_CEA_ were excised, and cryosections were costained with a rabbit polyclonal antiserum against *E*. *coli* (green) and a rat monoclonal antibody against murine CD105 (red). Cell nuclei were visualized by Hoechst (blue).
(PDF)

S7 Fig Characterization of the plasmid cured A30 strain (*E*. *coli* ΔAfaE-III). *(A)* Plasmids were isolated from *E*. *coli* AfaE-III wild type and the ΔAfaE-III strain and the non-restricted plasmid DNA was separated by electrophoresis. Two bands representing high molecular weight plasmid DNA found in wildtype A30 AfaE-III (red arrows) were absent in the ΔAfaE-III strain. PCR analysis verified that the *afa* locus is not present in the ΔAfaE-III strain, whereas the K5 capsule determinant as another virulence marker could be detected in both strains. *(B)* Growth of *E*. *coli* AfaE-III and *E*. *coli* ΔAfaE-III was monitored by OD600 readings over the course of 25h.
(PDF)

S8 Fig *E*. *coli* infection induces epithelial exfoliation in both TLR4+/+ and TLR4-/- mice. TLR4+/+(HeN) as well as TLR -/- (HeJ) female mice (n = 4) were infected with 1x10^6^ *E*. *coli* or left uninfected (uninf). After 24 h, whole mount urogenital tracts were dissected, fixed and processed for scanning electron microscopy. Pictures show the luminal surface of the upper vaginal and cervical regions. The boxed areas of the infected animals are enlarged in the lower panels to reveal details of epithelial exfoliation in both TLR4+/+ and TLR4-/- mice. Magnification as indicated by scale bars.
(PDF)
