# Supplementary material for: Uropathogenic E. coli Exploit CEA to Promote Colonization of the Urogenital Tract Mucosa
Source: PLoS Pathog. 2016 May 12;12(5):e1005608. doi: 10.1371/journal.ppat.1005608 (PMC4865239; doi:10.1371/journal.ppat.1005608)
Supplement: S1 Fig — (A) Lysates of E. coli, E. coli OpaCEA, and OpaCEA-expressing gonococci (Ngo OpaCEA) were analysed by Western blotting with a monoclonal anti-Opa antibody. (B) Growth of E. coli and E. coli OpaCEA in cell culture medium was monitored using optical density readings at 600 nm (OD600) every 60 min. Data plotted are the means of triplicate cultures. (C) Growth of E. coli OpaCEA in the presence or absence of IPTG was monitored as in (B). Data plotted are the means of triplicate cultures. (PDF) [file ppat.1005608.s001.pdf]

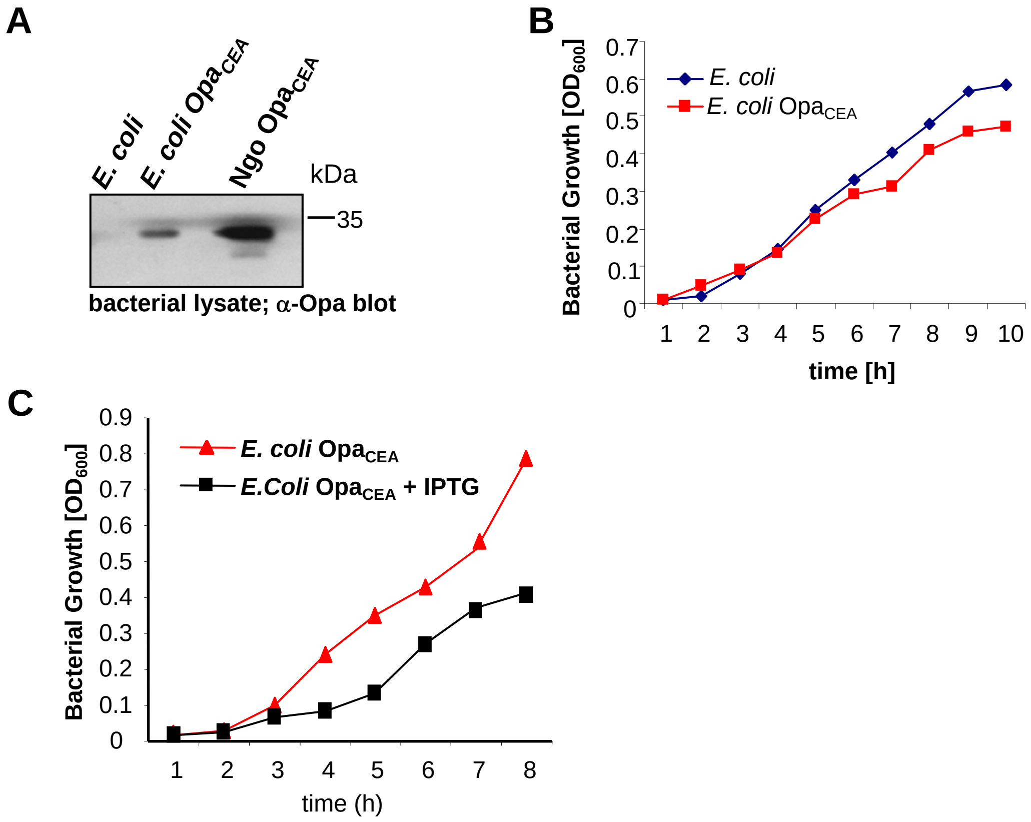

Figure S1. *E. coli* Opa$_{CEA}$ expresses the gonococcal Opa$_{CEA}$ adhesin and grows comparable to the *E. coli* control strain.
(A) Lysates of *E. coli*, *E. coli* Opa$_{CEA}$, and Opa$_{CEA}$-expressing gonococci (Ngo Opa$_{CEA}$) were analysed by Western blotting with a monoclonal anti-Opa antibody. *(B)* Growth of *E. coli* and *E. coli* Opa$_{CEA}$ in cell culture medium was monitored using optical density readings at 600 nm (OD_{600}) every 60 min. Data plotted are the means of triplicate cultures. *(C)* Growth of *E. coli* Opa$_{CEA}$ in the presence or absence of IPTG was monitored as in (B). Data plotted are the means of triplicate cultures.
