# Supplementary material for: Uropathogenic E. coli Exploit CEA to Promote Colonization of the Urogenital Tract Mucosa
Source: PLoS Pathog. 2016 May 12;12(5):e1005608. doi: 10.1371/journal.ppat.1005608 (PMC4865239; doi:10.1371/journal.ppat.1005608)
Supplement: S2 Fig — (A) ME-180 cells were analysed for endogenous CEACAMs by flow cytometry using monoclonal mAbs GM-8G5, recognizing CEACAM1 (red line), 9A6, recognizing CEACAM6 (red line), or COL-1, recognizing CEA (red line). Gray areas indicate staining of cells with isotype-matched control antibodies. (B) ME-180 cells were seeded on glasscover slips, infected or not with the indicated bacteria for 2 h with an MOI of 20, fixed and stained with antibodies against endogenous CEACAMs using clone D14HD11 (red) and rabbit -E. coli (green) or -N. gonorrhoeae (green). Bacteria bound to the CEA-positive cells are indicated by arrowheads. (PDF) [file ppat.1005608.s002.pdf]

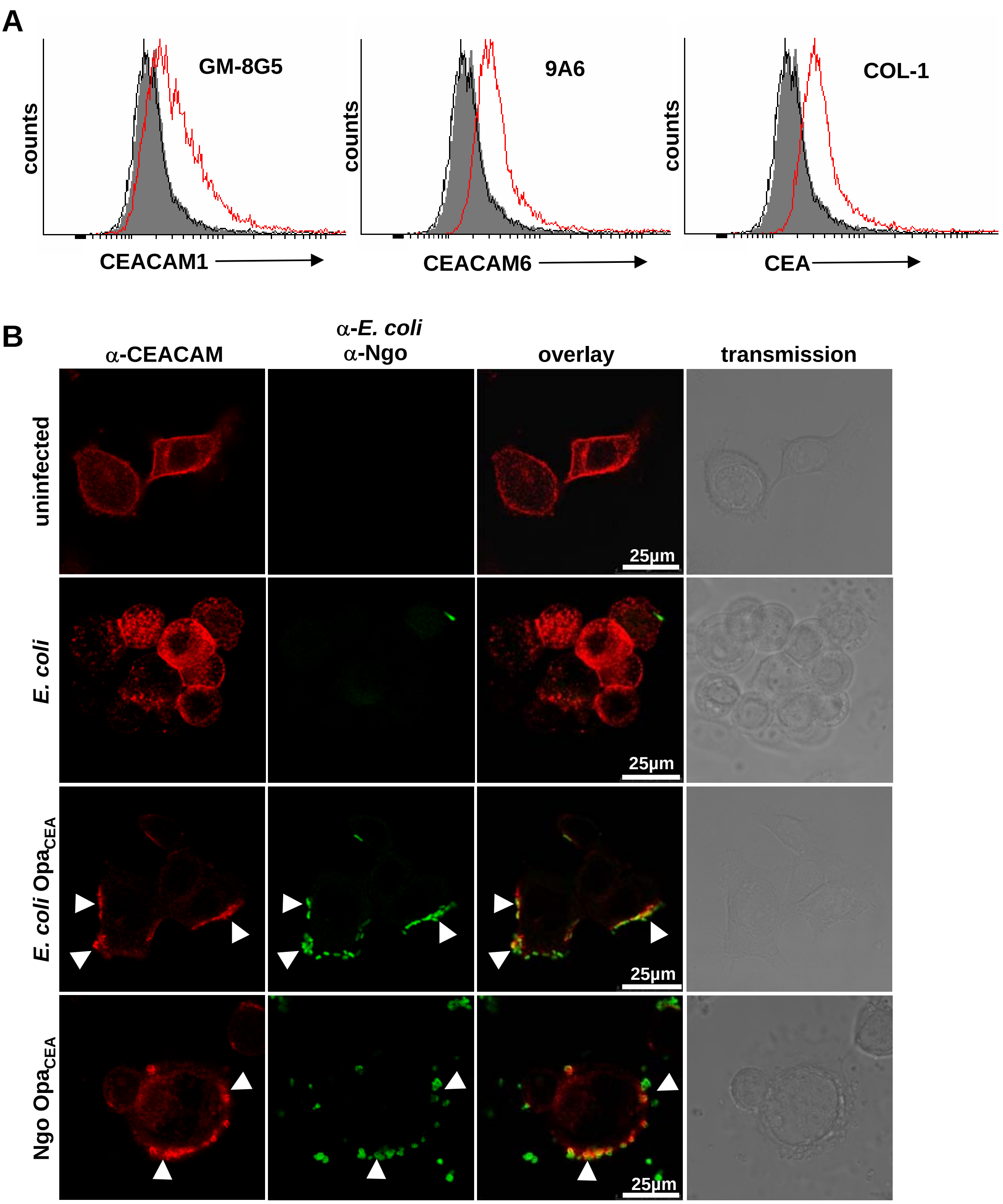

Figure S2. ***E. coli* Opa triggers recruitment of CEACAMs**

(A) ME-180 cells were analysed for endogenous CEACAMs by flow cytometry using monoclonal mAbs GM-8G5, recognizing CEACAM1 (red line), 9A6, recognizing CEACAM6 (red line), or COL-1, recognizing CEA (red line). Gray areas indicate staining of cells with isotype-matched control antibodies. *(B)* ME-180 cells were seeded on glasscover slips, infected or not with the indicated bacteria for 2 h with an MOI of 20, fixed and stained with antibodies against endogenous CEACAMs using clone D14HD11 (red) and rabbit α-*E. coli* (green) or α-*N. gonorrhoeae* (green). Bacteria bound to the CEA-positive cells are indicated by arrowheads.
