# Supplementary material for: Uropathogenic E. coli Exploit CEA to Promote Colonization of the Urogenital Tract Mucosa
Source: PLoS Pathog. 2016 May 12;12(5):e1005608. doi: 10.1371/journal.ppat.1005608 (PMC4865239; doi:10.1371/journal.ppat.1005608)
Supplement: S3 Fig — (A) Human vaginal epithelial cells (hVECs) cells were seeded in 24-well plates coated with 25 μg/ml collagen. Confluent layers were left uninfected or infected for 14 h with E. coli; E. coli OpaCEA; piliated, non-opaque N. gonorrhoeae (Ngo P+); non-piliated gonococci expressing a heparansulphate proteoglycan-binding Opa protein (Ngo OpaHSPG); or non-piliated gonococci expressing a CEACAM-binding Opa protein (Ngo OpaCEA). Following infection, cells were washed and remaining cells were stained with crystal violet. Representative areas with remaining cells were photographed. (B) hVEC cells were infected and stained as in (A). Staining intensity of undetached cells was determined after dye elution in a spectrophotometer at 550 nm. Bars represent mean ± S.D. of 6 wells. (C) hVECs were analysed for CEACAM expression by flow cytometry using a mouse monoclonal anti-CEACAM antibody (clone D14HD11; red line). Gray area indicates staining of hVECs with isotype-matched control antibody. (PDF) [file ppat.1005608.s003.pdf]

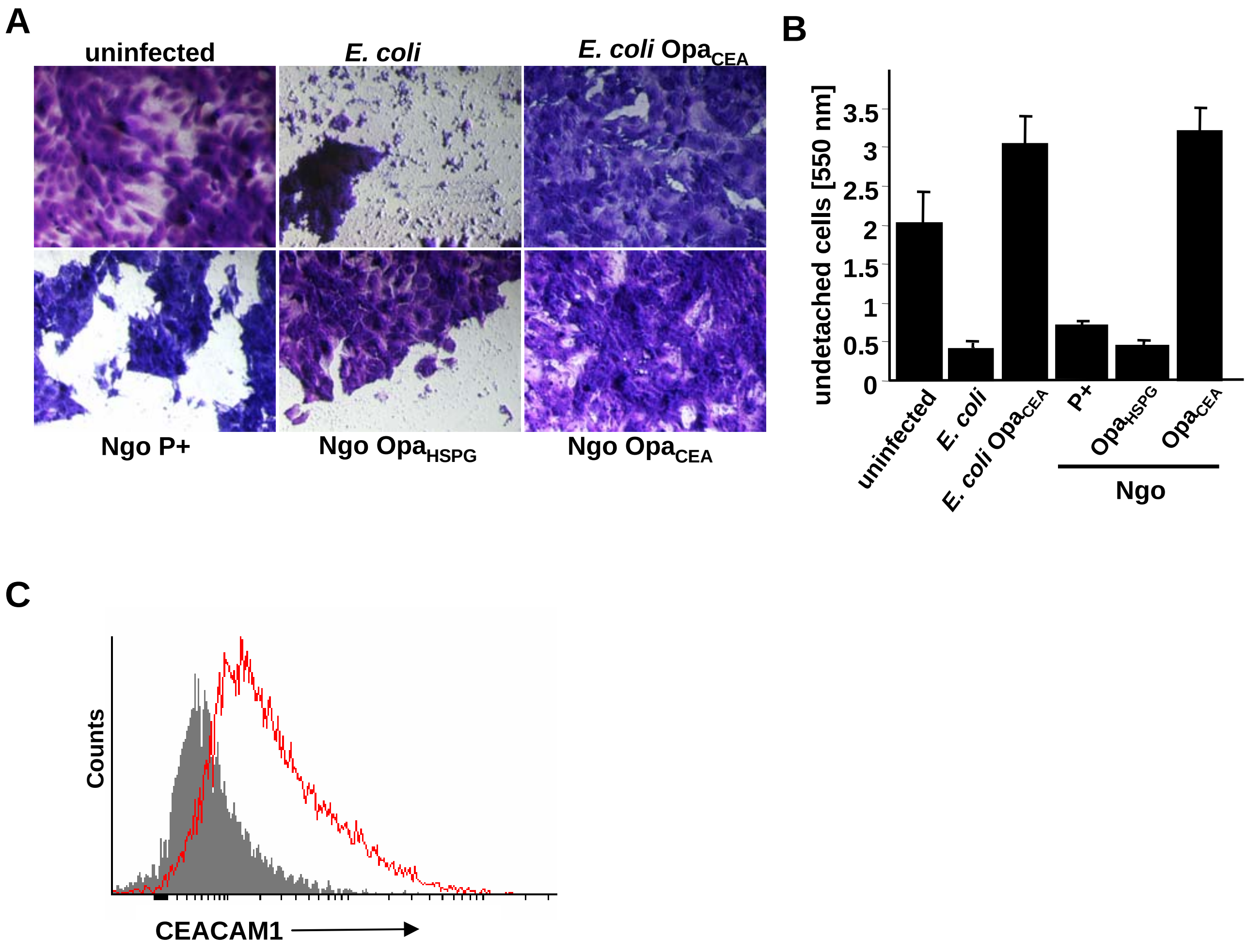

Figure S3. ***E. coli* Opa$_{CEA}$ suppresses detachment of primary vaginal epithelial cells.** *(A)* Human vaginal epithelial cells (hVECs) cells were seeded in 24-well plates coated with 25 µg/ml collagen. Confluent layers were left uninfected or infected for 14 h with *E. coli*; *E. coli* Opa$_{CEA}$; piliated, non-opaque *N. gonorrhoeae* (Ngo P+); non-piliated gonococci expressing a heparansulphate proteoglycan-binding Opa protein (Ngo Opa$_{HSPG}$); or non-piliated gonococci expressing a CEACAM-binding Opa protein (Ngo Opa$_{CEA}$). Following infection, cells were washed and remaining cells were stained with crystal violet. Representative areas with remaining cells were photographed. *(B)* hVEC cells were infected and stained as in (A). Staining intensity of undetached cells was determined after dye elution in a spectrophotometer at 550 nm. Bars represent mean ± S.D. of 6 wells. *(C)* hVECs were analysed for CEACAM expression by flow cytometry using a mouse monoclonal anti-CEACAM antibody (clone D14HD11; red line). Gray area indicates staining of hVECs with isotype-matched control antibody.
