# Supplementary material for: Uropathogenic E. coli Exploit CEA to Promote Colonization of the Urogenital Tract Mucosa
Source: PLoS Pathog. 2016 May 12;12(5):e1005608. doi: 10.1371/journal.ppat.1005608 (PMC4865239; doi:10.1371/journal.ppat.1005608)
Supplement: S4 Fig — (A) 293 cells were transiently transfected with an empty control plasmid or a CEA-encoding plasmid and analysed by flow cytometry. About ~40% of the cell population showed CEA surface expression after transfection as detected by a monoclonal CEACAM antibody. Gray area indicates staining of CEA-transfected cells with an isotype matched control antibody. (B) 293 cells were transfected with the empty vector control (pcDNA) or plasmids encoding CEA or CD105. Cells were either left uninfected or infected for 8 h with the indicated bacteria. Then, cells were used in adhesion assays on collagen. Bars represent means ± SD of eight samples. Two-tailed student’s t-test; *** p < 0.001. (C) 293T cells transfected with a CEA-encoding plasmid were either left uninfected or infected for 14 h with E. coli, E. coli OpaCEA, or Ngo OpaCEA and analyzed by flow cytometry with a monoclonal anti-human CD105 antibody. Gray area indicates staining of uninfected cells. (PDF) [file ppat.1005608.s004.pdf]

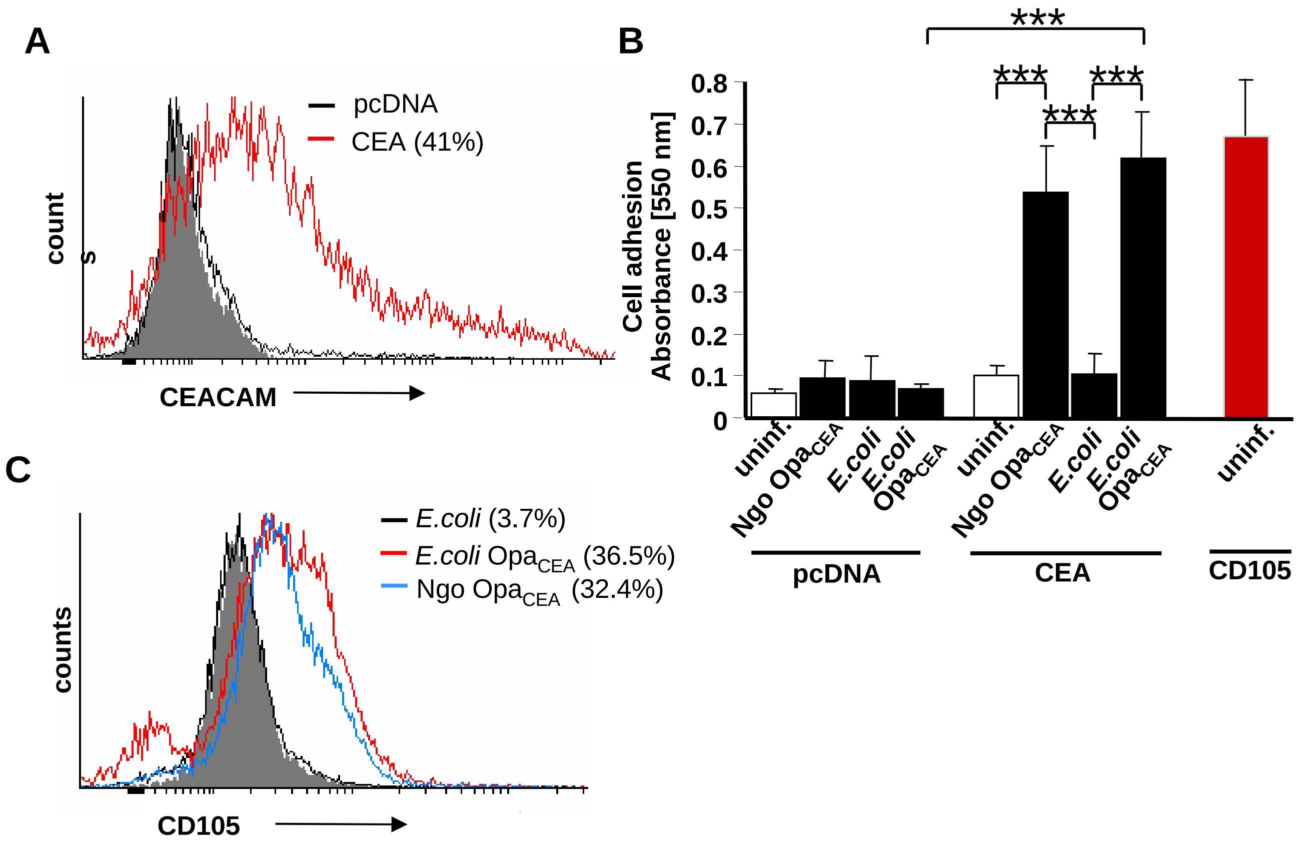

Figure S4. CEA binding by *E. coli* is accompanied by increased cell-matrix adhesion and upregulation of CD105.

(A) 293 cells were transiently transfected with an empty control plasmid or a CEA-encoding plasmid and analysed by flow cytometry. About ~40% of the cell population showed CEA surface expression after transfection as detected by a monoclonal CEACAM antibody. Gray area indicates staining of CEA-transfected cells with an isotype matched control antibody. *(B)* 293 cells were transfected with the empty vector control (pcDNA) or plasmids encoding CEA or CD105. Cells were either left uninfected or infected for 8 h with the indicated bacteria. Then, cells were used in adhesion assays on collagen. Bars represent means ± SD of eight samples. Two-tailed student's t-test; *** $p < 0.001$. *(C)* 293T cells transfected with a CEA-encoding plasmid were either left uninfected or infected for 14 h with *E. coli*, *E. coli* Opa$_{CEA}$, or Ngo Opa$_{CEA}$ and analyzed by flow cytometry with a monoclonal anti-human CD105 antibody. Gray area indicates staining of uninfected cells.
