# Supplementary material for: Uropathogenic E. coli Exploit CEA to Promote Colonization of the Urogenital Tract Mucosa
Source: PLoS Pathog. 2016 May 12;12(5):e1005608. doi: 10.1371/journal.ppat.1005608 (PMC4865239; doi:10.1371/journal.ppat.1005608)
Supplement: S5 Fig — (A, B) 293 cells were transiently transfected with plasmids encoding either CEA or CD105. Transfected cells were infected with indicated bacteria for 14 h or left uninfected. Next, cells were replated onto collagen-coated culture dishes for 90 min and stimulated or not for 5 min with 1 mM Mn2+ before fixation. Fixed samples were either stained with a rat monoclonal integrin 1 antibody (clone AIIB2), which recognizes the integrin 1 extracellular domain irrespective of its conformation (total integrin 1) (A) or samples were stained with an activation-epitope specific rat monoclonal integrin 1 antibody (clone 9EG7), which recognizes the extended, ligand-bound conformation of integrin 1 (active integrin 1) (B). Bars represent the mean ± s.d. of 5 wells of a representative experiment repeated twice with similar results. (C, D) 293 cells were transiently transfected with the empty control vector (pcDNA) or a plasmid encoding CD105 and infected as indicated. Total integrin 1 and active integrin 1 (clone 9EG7) were detected as in (A, B). Bars represent the mean ± s.d. of 5 wells of a representative experiment repeated twice with similar results. Two-tailed student’s t-test; *** p < 0.001, n.s.—not significant. (PDF) [file ppat.1005608.s005.pdf]

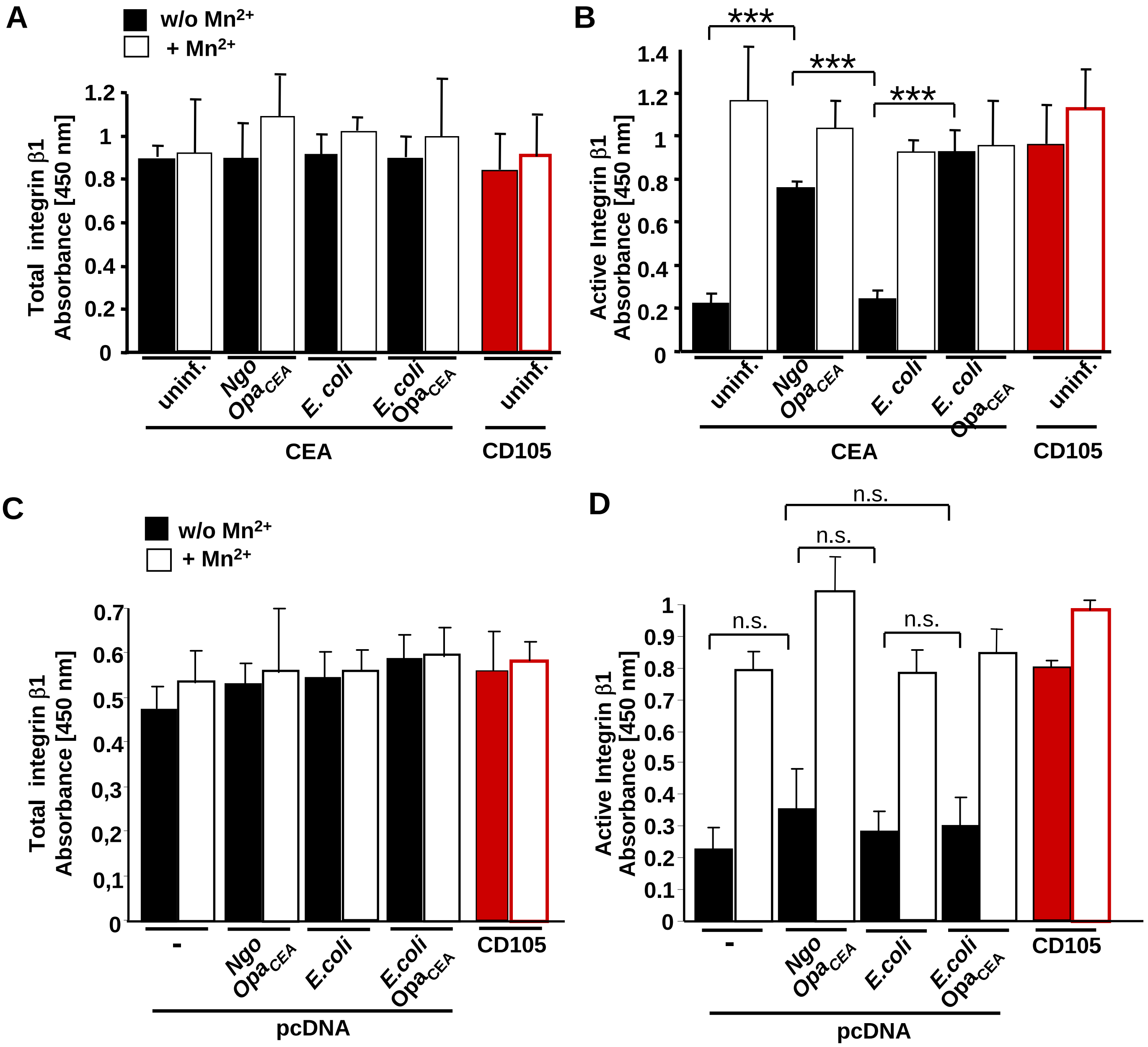

Figure S5. *E. coli* Opa$_{CEA}$ trigger enhanced integrin activity via CEACAM stimulation. *(A, B)* 293 cells were transiently transfected with plasmids encoding either CEA or CD105. Transfected cells were infected with indicated bacteria for 14 h or left uninfected. Next, cells were replated onto collagen-coated culture dishes for 90 min and stimulated or not for 5 min with 1 mM Mn^{2+} before fixation. Fixed samples were either stained with a rat monoclonal integrin β1 antibody (clone AIIB2), which recognizes the integrin β1 extracellular domain irrespective of its conformation (total integrin β1) (A) or samples were stained with an activation-epitope specific rat monoclonal integrin β1 antibody (clone 9EG7), which recognizes the extended, ligand-bound conformation of integrin β1 (active integrin β1) (B). Bars represent the mean ± s.d. of 5 wells of a representative experiment repeated twice with similar results. *(C, D)* 293 cells were transiently transfected with the empty control vector (pcDNA) or a plasmid encoding CD105 and infected as indicated. Total integrin β1 and active integrin β1 (clone 9EG7) were detected as in (A, B). Bars represent the mean ± s.d. of 5 wells of a representative experiment repeated twice with similar results. Two-tailed student's t-test; *** $p < 0.001$, n.s. - not significant.
