# Supplementary material for: Uropathogenic E. coli Exploit CEA to Promote Colonization of the Urogenital Tract Mucosa
Source: PLoS Pathog. 2016 May 12;12(5):e1005608. doi: 10.1371/journal.ppat.1005608 (PMC4865239; doi:10.1371/journal.ppat.1005608)
Supplement: S6 Fig — Genital tracts from wild-type mice infected for 24 hours with E. coli or E. coli OpaCEA were excised, and cryosections were costained with a rabbit polyclonal antiserum against E. coli (green) and a rat monoclonal antibody against murine CD105 (red). Cell nuclei were visualized by Hoechst (blue). (PDF) [file ppat.1005608.s006.pdf]

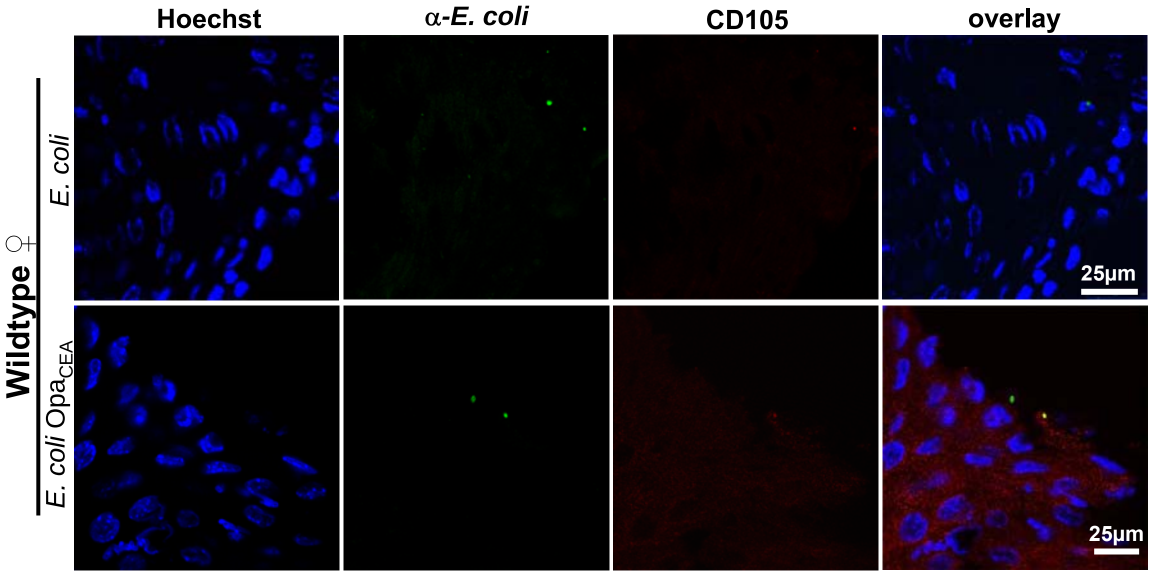

Figure S6. *E. coli* Opa$_{CEA}$ does not induce CD105 expression in wildtype animals. Genital tracts from wild-type mice infected for 24 hours with *E. coli* or *E. coli* Opa$_{CEA}$ were excised, and cryosections were costained with a rabbit polyclonal antiserum against *E. coli* (green) and a rat monoclonal antibody against murine CD105 (red). Cell nuclei were visualized by Hoechst (blue).
