# Supplementary material for: Uropathogenic E. coli Exploit CEA to Promote Colonization of the Urogenital Tract Mucosa
Source: PLoS Pathog. 2016 May 12;12(5):e1005608. doi: 10.1371/journal.ppat.1005608 (PMC4865239; doi:10.1371/journal.ppat.1005608)
Supplement: S7 Fig — (A) Plasmids were isolated from E. coli AfaE-III wild type and the ΔAfaE-III strain and the non-restricted plasmid DNA was separated by electrophoresis. Two bands representing high molecular weight plasmid DNA found in wildtype A30 AfaE-III (red arrows) were absent in the ΔAfaE-III strain. PCR analysis verified that the afa locus is not present in the ΔAfaE-III strain, whereas the K5 capsule determinant as another virulence marker could be detected in both strains. (B) Growth of E. coli AfaE-III and E. coli ΔAfaE-III was monitored by OD600 readings over the course of 25h. (PDF) [file ppat.1005608.s007.pdf]

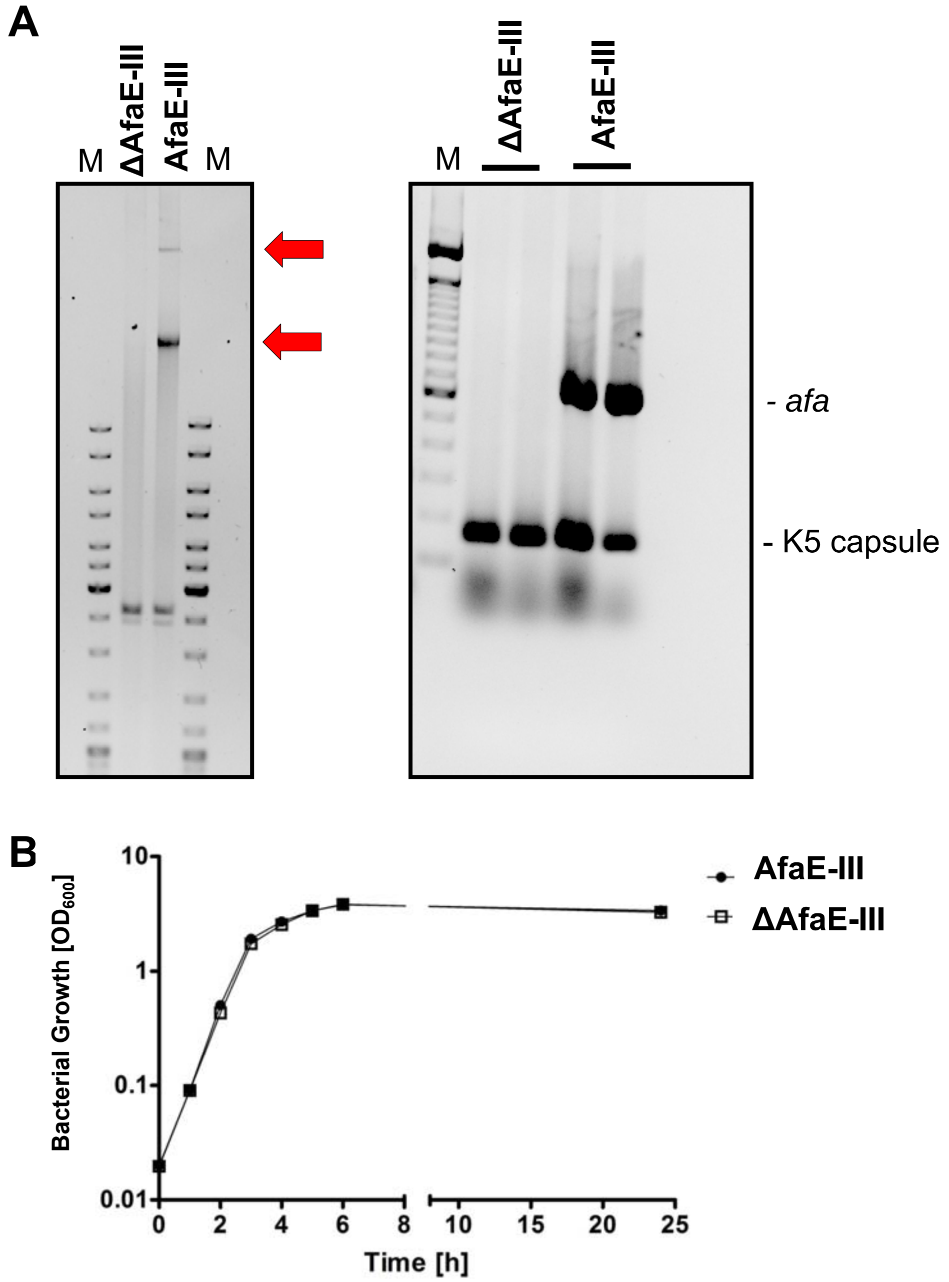

Figure S7. ***Characterization of the plasmid cured A30 strain (E. coli ΔAfaE-III).***
(A) Plasmids were isolated from *E. coli* AfaE-III wild type and the ΔAfaE-III strain and the non-restricted plasmid DNA was separated by electrophoresis. Two bands representing high molecular weight plasmid DNA found in wildtype A30 AfaE-III (red arrows) were absent in the ΔAfaE-III strain. PCR analysis verified that the *afa* locus is not present in the ΔAfaE-III strain, whereas the K5 capsule determinant as a chromosomally encoded virulence marker could be detected in both strains. *(B)* Growth of *E. coli* AfaE-III and *E. coli* ΔAfaE-III was monitored by OD600 readings over the course of 25h.
