# Supplementary material for: Uropathogenic E. coli Exploit CEA to Promote Colonization of the Urogenital Tract Mucosa
Source: PLoS Pathog. 2016 May 12;12(5):e1005608. doi: 10.1371/journal.ppat.1005608 (PMC4865239; doi:10.1371/journal.ppat.1005608)
Supplement: S8 Fig — TLR4+/+(HeN) as well as TLR -/- (HeJ) female mice (n = 4) were infected with 1x10^6^ E. coli or left uninfected (uninf). After 24 h, whole mount urogenital tracts were dissected, fixed and processed for scanning electron microscopy. Pictures show the luminal surface of the upper vaginal and cervical regions. The boxed areas of the infected animals are enlarged in the lower panels to reveal details of epithelial exfoliation in both TLR4+/+ and TLR4-/- mice. Magnification as indicated by scale bars. (PDF) [file ppat.1005608.s008.pdf]

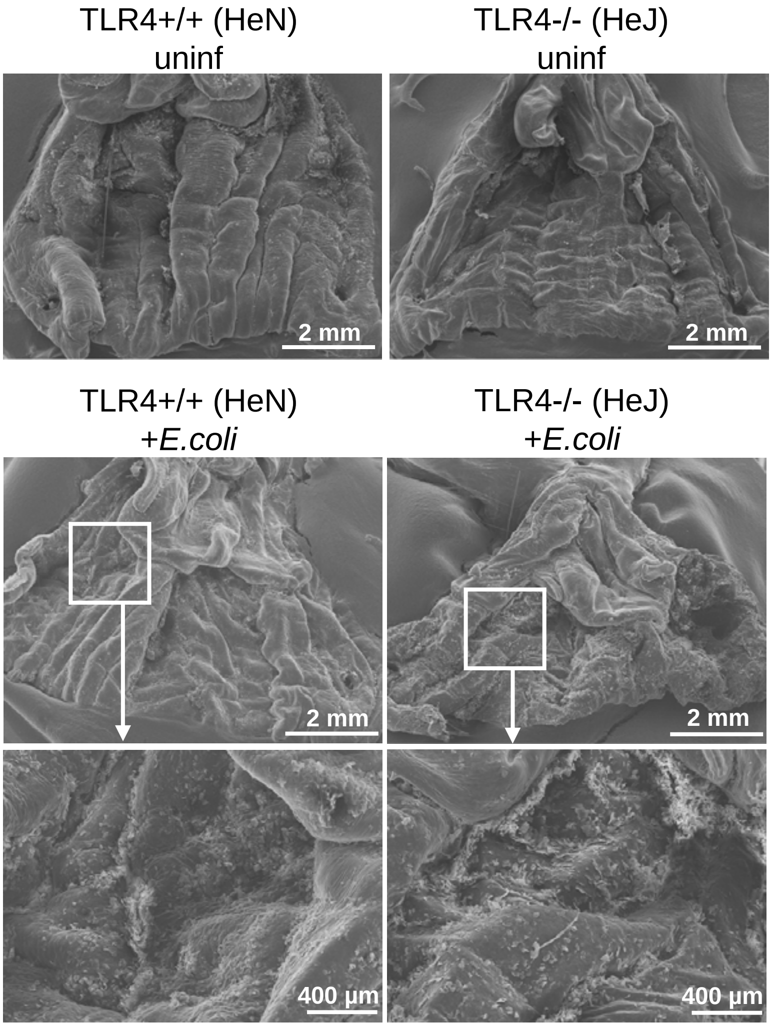


**Figure S8. *E. coli* infection induces epithelial exfoliation in both TLR4+/+ and TLR4-/- mice.** TLR4+/+(HeN) as well as TLR -/- (HeJ) female mice (n = 4) were infected with $1x10^6$ *E. coli* or left uninfected (uninf). After 24 h, whole mount urogenital tracts were dissected, fixed and processed for scanning electron microscopy. Pictures show the luminal surface of the upper vaginal and cervical regions. The boxed areas of the infected animals are enlarged in the lower panels to reveal details of epithelial exfoliation in both TLR4+/+ and TLR4-/- mice. Magnification as indicated by scale bars.
